# Conditional mutagenesis by oligonucleotide-mediated integration of loxP sites in zebrafish

**DOI:** 10.1371/journal.pgen.1007754

**Published:** 2018-11-14

**Authors:** Leonard Burg, Nicholas Palmer, Khrievono Kikhi, Evgeniya S. Miroshnik, Helen Rueckert, Eleanor Gaddy, Carlee MacPherson Cunningham, Kenny Mattonet, Shih-Lei Lai, Rubén Marín-Juez, Richard B. Waring, Didier Y. R. Stainier, Darius Balciunas

**Affiliations:** 1 Department of Biology, College of Science and Technology, Temple University, Philadelphia, Pennsylvania, United States of America; 2 Department of Developmental Genetics, Max Planck Institute for Heart and Lung Research, Bad Nauheim, Germany; University of Pennsylvania School of Medicine, UNITED STATES

## Abstract

Many eukaryotic genes play essential roles in multiple biological processes in several different tissues. Conditional mutants are needed to analyze genes with such pleiotropic functions. In vertebrates, conditional gene inactivation has only been feasible in the mouse, leaving other model systems to rely on surrogate experimental approaches such as overexpression of dominant negative proteins and antisense-based tools. Here, we have developed a simple and straightforward method to integrate loxP sequences at specific sites in the zebrafish genome using the CRISPR/Cas9 technology and oligonucleotide templates for homology directed repair. We engineered conditional (floxed) mutants of *tbx20* and *fleer*, and demonstrate excision of exons flanked by loxP sites using tamoxifen-inducible CreERT2 recombinase. To demonstrate broad applicability of our method, we also integrated loxP sites into two additional genes, *aldh1a2* and *tcf21*. The ease of this approach will further expand the use of zebrafish to study various aspects of vertebrate biology, especially post-embryonic processes such as regeneration.

## Author summary

Some genes are expressed and function in a single tissue, and the effect of their loss on that tissue can be readily determined. Frequently, however, genes that are necessary for the development or maintenance of one tissue are also important for other tissues or cell types. Genes of the latter type are difficult to analyze because of the complications resulting from an organism having multiple defects in different tissues. The solution pioneered by mouse geneticists is to inactivate the gene of interest in only one tissue at a time. This elegant approach requires the ability to make specific edits to the genome, a technology that was not readily available to zebrafish researchers until recently. Using the CRISPR/Cas9 genome editing tool, we have developed a simple, reliable, and efficient method to insert DNA sequences into the zebrafish genome that enable conditional gene inactivation. Our methodology will be useful not only for the study of genes that play important roles in multiple tissues, but also for the genetic analysis of biological processes which occur in adult animals.

## Introduction

Model system genetics is undergoing a major shift from forward to reverse genetics, driven by a combination of two key factors. The first is the increasing robustness of genomic and proteomic tools, which enable rapid identification of candidate genes with potential roles in biological processes of interest. The second factor is the proliferation of precise, efficient, and easy to use genome editing tools, from zinc finger nucleases to TALE-guided nucleases and CRISPR/Cas9 (reviewed in [1]). CRISPR/Cas9 in particular has made introduction of mutations into genomes of various organisms very straightforward and cost-effective.

Zebrafish first rose to prominence as a vertebrate model system that made development and organogenesis amenable to forward genetic analyses [2, 3]. Over the past twenty years, use of the zebrafish has expanded to include biological processes which occur much later in ontogenesis. Examples of such biological processes include regeneration [4], various aspects of behavior from habituation [5] to sleep-wake cycle [6], and carcinogenesis [7].

Genetic analyses of post-embryonic processes in zebrafish have had to rely on overexpression of dominant negatives, knockdown using morpholino oligonucleotides, modulation using small molecules, and similar approaches. The inability to generate true conditional mutants has led scientists to try alternatives, such as mutant rescue using floxed Bacterial Artificial Chromosomes [8], random gene trapping using conditional vectors [9–11], or targeted integration of a Cre-inducible gene trap cassette [12]. Only recently has generation of the first floxed zebrafish mutant been reported [13]. In contrast, mouse geneticists have been refining conditional mutagenesis since the introduction of the term “floxed” in 1994 [14].

We have recently developed a method for integration of epitope-coding sequences into the zebrafish genome using oligonucleotide-directed repair of CRISPR/Cas9-induced double strand breaks [15]. High germline transmission rates suggested that sequential integration of two loxP sites might be feasible. Single-stranded oligonucleotide templates have been successfully used to integrate loxP sites, either sequentially or two at a time, into the mouse genome [16–19]. However, since only non-palindromic mlox sites have so far been successfully integrated into the zebrafish genome using oligonucleotide templates [20, 21], we were concerned that the palindromic nature of the loxP site may interfere with homology-directed repair in zebrafish embryos.

To test the feasibility of oligonucleotide-mediated integration of loxP sites, we selected four genes which play essential roles in development and may also be required for regeneration: *tbx20*, *fleer*, *aldh1a2* and *tcf21*. *tbx20* is required for heart development in mouse and zebrafish, but is also required for mouse cardiac homeostasis [22–26]. *tbx20* is upregulated during zebrafish heart regeneration, and overexpression of Tbx20 improves the regenerative capacity of the mouse heart [27, 28]. *fleer* is essential for ciliogenesis, and zebrafish embryos lacking *fleer* display pleiotropic phenotypes including laterality defects, kidney cysts, and failure to inflate brain ventricles [29]. While it is not known if cilia are required for regeneration, their roles in mechanosensing as well as *shh* signal transduction suggest that they may be needed for regeneration to occur. *aldh1a2* mutants display pleiotropic phenotypes in neural, heart, and pectoral fin development [30, 31]. Upregulation of *aldh1a2* mRNA is the signature of endocardial and epicardial activation after cardiac injury [32]. Finally, *tcf21* mutants display defects in morphogenesis of branchial arch-derived structures [15, 33]. *tcf21* is also constitutively expressed in the epicardium and epicardial-derived cells of adult zebrafish, and *tcf21* expressing cells are required for cardiac regeneration [34].

Here we found that single stranded oligonucleotide templates can efficiently direct integration of loxP sites into CRISPR/Cas9-induced double strand breaks in the zebrafish genome. We performed sequential integration of two loxP sites to generate a conditional (floxed) allele of *tbx20*. We have used this conditional allele to demonstrate that early expression *tbx20* is essential for zebrafish heart development. Using *fleer* as a model, we demonstrate that Cre-revertible Gene Breaking Transposon (GBT) mutants can be readily converted into fully conditional alleles by the addition of a single loxP site. Using *aldh1a2*, we demonstrate that prior to -or in parallel with- generation of a floxed allele, the target exon can be readily removed to test if the deletion produces a phenotype. Finally, using *tcf21* as a model for small genes, we demonstrate that a loxP site can be integrated into the 5’ untranslated region without significantly impairing gene function.

## Results

### Efficient oligonucleotide-mediated integration of loxP sites into the zebrafish genome

**Fig 1:**
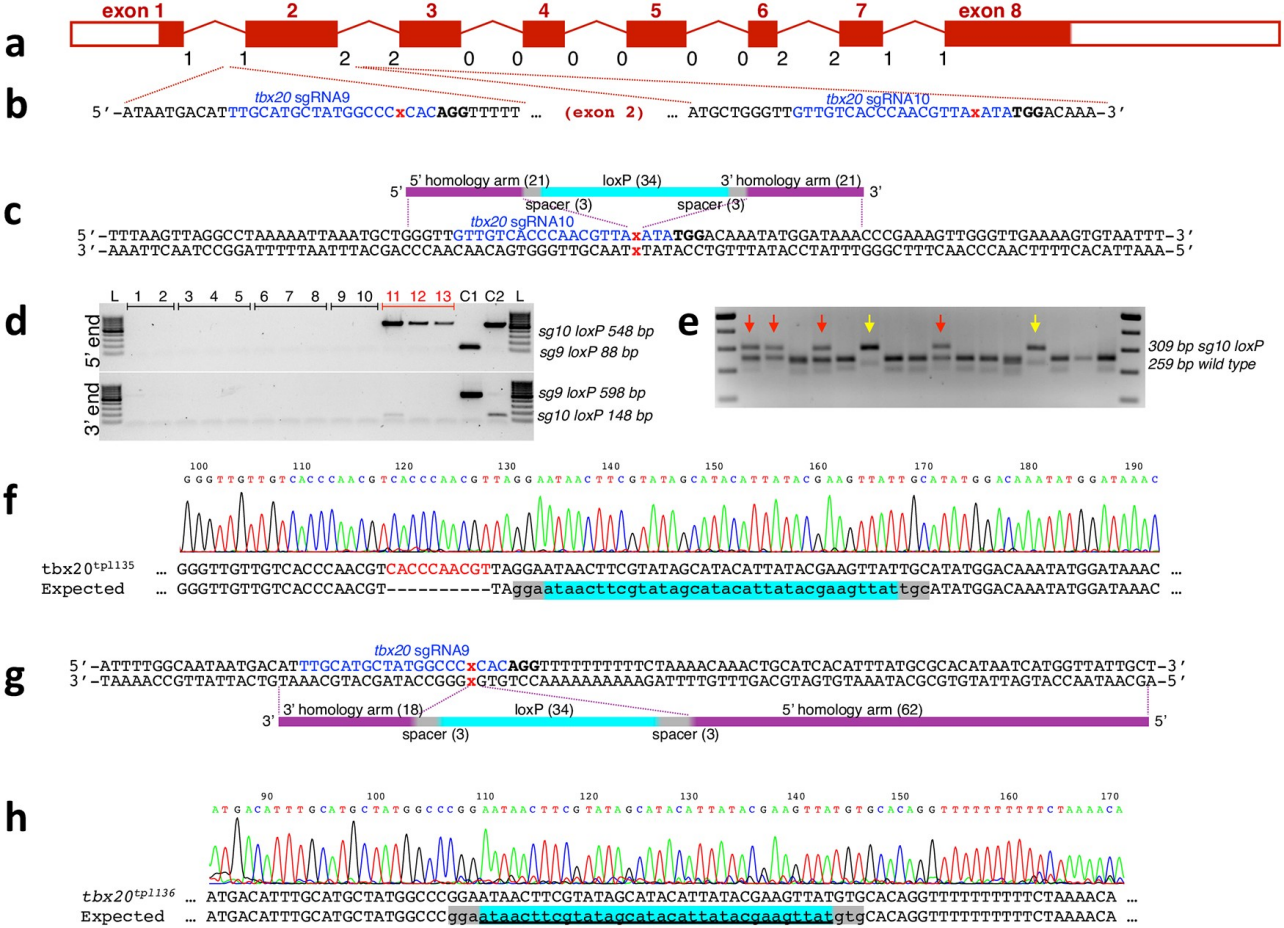
Efficient integration of loxP sites into the first and second introns of *tbx20*. **a**. Diagram of the *tbx20* locus. Exons are drawn to scale, introns not to scale. The number below each intron-exon junction indicates reading frame phase. **b**. Target sites of two highly active intronic *tbx20* sgRNAs, *tbx20* sgRNA9 and *tbx20* sgRNA10, flanking exon 2. Sequence corresponding to single guide RNA is shown in blue, PAM motif is in bold, and expected Cas9 cut site is indicated by a red x. **c-f**. Integration of loxP site into intron 2 of *tbx20*. **c**. The single stranded oligonucleotide used for homology-directed repair was in sense strand with regard to the PAM and had loxP (aqua) site flanked by 3 nucleotide spacer sequences (grey), and 21 nucleotide long homology arms. **d**. Representative image of the 5’ nested PCR reaction used to screen pools of F1 embryos. L, 100 bp DNA ladder (ThermoFisher Scientific). 1–13, PCR on pools of embryos from 5 different F1 in-crosses (pools from the same F0 pair are grouped, loxP-positive cross is underlined in red). C1 control DNA from embryos injected with *tbx20* sgRNA9 and *tbx20*sg9-loxP HDR oligonucleotide. C2, control DNA from embryos injected with *tbx20* sgRNA10 and *tbx20*sg10-loxP HDR oligonucleotide. **e**. PCR genotyping of tail clips of adult F1 fish from the F0 in-cross underlined in red in **d**. Red arrows indicate fish heterozygous for the loxP-containing allele. Yellow arrows indicate fish which likely contain one loxP-containing allele, but presumably lack the wild type allele due to a deletion inherited from the other parent. **f**. Sequence of the recovered *tpl135* allele containing integration of the full-length loxP site with 10-nucleotide partial target site duplication at the 5’ of the HDR oligonucleotide. **g, h**. Experimental design for integration of loxP site into intron 1 of *tbx20*. **g**. The single stranded oligonucleotide used for homology-directed repair was antisense to the PAM-containing strand and had loxP site flanked by 3-nucleotide spacer sequences, 62-nucleotide 5’ homology arm and 18-nucleotide 3’ homology arm. **h**. Sequence of the recovered *tpl136* allele containing perfect integration of the loxP site.

Conditional loss-of-function mutagenesis requires two loxP sites flanking an exon (or several exons) of a gene. We have recently developed a methodology for oligonucleotide-mediated integration of epitope-coding sequences into the zebrafish genome [15]. The relatively high efficiency of our method prompted us to speculate that conditional mutagenesis could be achieved by sequential integration of loxP sites. However, since oligonucleotide-mediated integration of wild type loxP sites into the zebrafish genome has not yet been demonstrated, we first needed to test if it was at all feasible. We selected *tbx20* for our initial experiments (Fig 1) and chose to flox its second exon because it encodes the first few amino acids of the T-box DNA binding domain, and because removal of the second exon would put exons 3–7 out of reading frame. Two highly active sgRNAs, *tbx20* sgRNA9 and *tbx20* sgRNA10 (Fig 1a and 1b), flanking the second exon, were identified by loss of a restriction enzyme site and/or T7 endonuclease assay (assessment of *tbx20*sgRNA9 activity is shown in S1 Fig).

To integrate a loxP site into intron 2 of *tbx20*, we designed an oligonucleotide template for homology directed repair of the double strand break induced by *tbx20* sgRNA10. (Fig 1c). Since we have frequently observed small indels at the homology arm junction in past experiments, we decided to flank the loxP sequence with three nucleotides of “spacer” sequence. We injected *tbx20* sgRNA10 along with nCas9n mRNA into embryos at the one cell stage, followed by injection of the HDR oligonucleotide with 21 nucleotide-long homology arms as previously described [15]. Indels were readily detected using the T7 endonuclease assay, and integration of the loxP site was detected by PCR in pooled injected embryos. Siblings of tested embryos were raised, incrossed, and screened for germline transmission of integration of the loxP site by nested PCR. One out of twelve incrosses produced embryos positive by both 5’ and 3’ nested PCR. Notably, nested PCR for the 3’ end worked poorly, with only one of three batches of embryos appearing barely positive (Fig 1d top vs bottom panels). Poor amplification with loxP-specific primers turned out to be a recurring issue. Several different loxP primers were designed over the course of this work; for some loci, several loxP-specific primers were tested on injected embryos. Nevertheless, sequencing of the 5’ and 3’ PCR fragments indicated integration of the full-length loxP site, with a 10-nucleotide partial target site duplication at the 5’ end of the HDR oligonucleotide template. Integration of the loxP site was further confirmed by performing a short flanking PCR on individual embryos and sequencing the larger PCR fragment. Siblings were raised to adulthood and 16 were genotyped by tail clip. We identified four adults heterozygous for a loxP site (Fig 1e, red arrows, sequenced in Fig 1f). Two additional fish had a single band corresponding to a loxP-containing allele, but no wild type band (yellow arrows). Since both parents were mutagenized, we interpreted this as the presence of a deletion removing at least one of the primer binding sites on the homologous chromosome.

In parallel, we performed experiments to integrate a loxP site into the first intron of *tbx20*. Sequence analysis revealed the presence of a polymorphic poly-T stretch a few nucleotides downstream of *tbx20* sgRNA9 target site, with wild type AB fish having either 9 or 10 thymidines. The shorter stretch (9 thymidines) seemed more prevalent among the fish we were injecting. In this experiment, we tested an HDR oligonucleotide template with asymmetric-length homology arms, antisense to the PAM-containing strand [35]. We designed a 120 nt-long oligonucleotide template for HDR (Fig 1g), and injected it into 1-cell zebrafish embryos along with nCas9n mRNA and *tbx20*sgRNA9 RNA. Integration of the loxP site was detected in pools as well as in individual injected embryos as described in Materials and Methods. Siblings of tested embryos were raised to adulthood and screened for germline transmission of a loxP site by nested PCR as described in Materials and Methods. One out of three incrosses gave embryos that were positive for the loxP site both at the 5’ and 3’end by nested PCR. Integration of the loxP site was confirmed by sequencing individual embryos. Siblings of genotyped embryos were raised to adulthood and 16 fish were genotyped for presence of the loxP site by short flanking PCR. Three fish were found to be heterozygous for the loxP-containing allele, leading to establishment of the *tbx20*^*tpl136*^ loxP integration line (Fig 1h).

### Generation of a conditional allele by sequential integration of loxP sites

**Fig 2:**
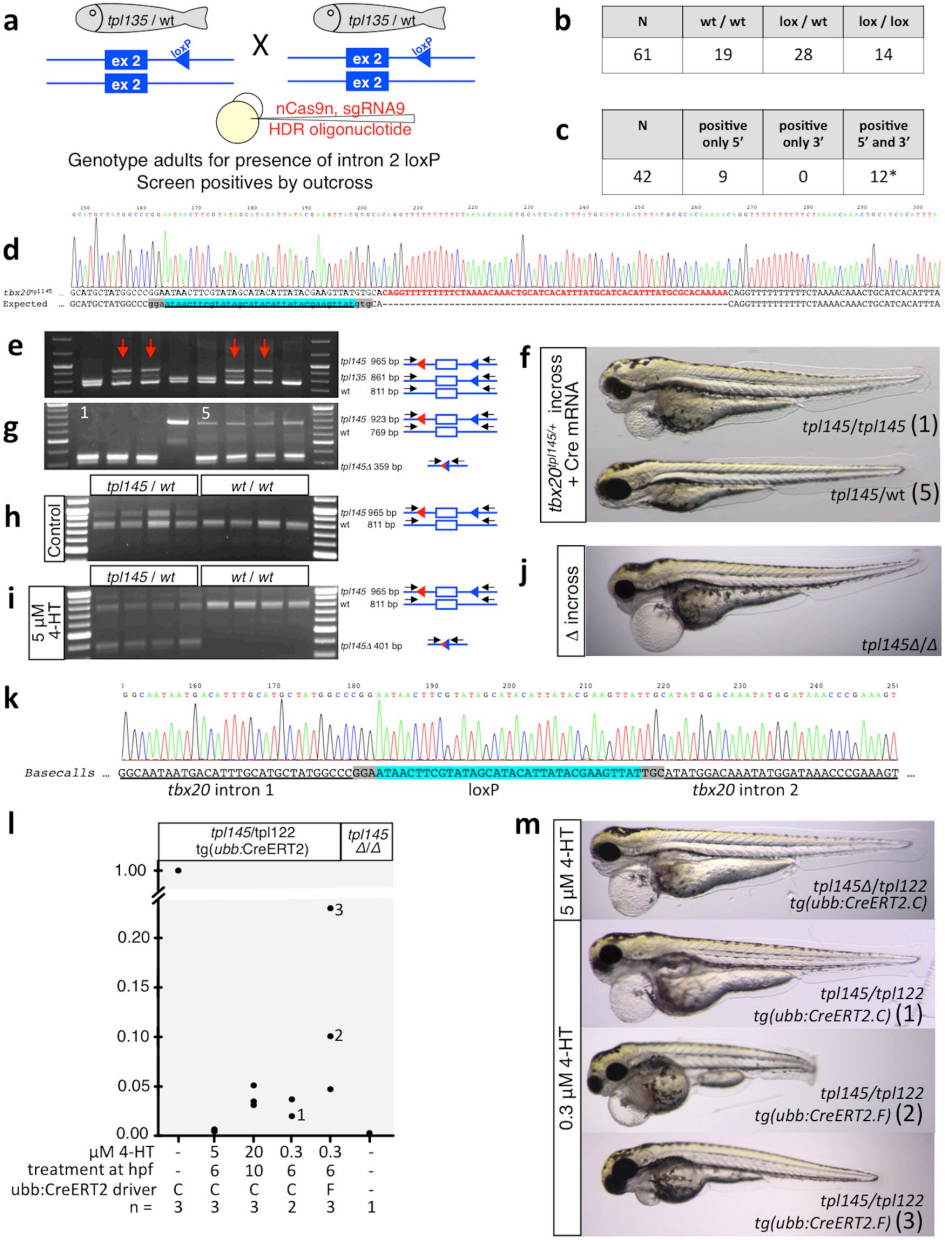
Generation and testing of a conditional (“floxed”) *tbx20* allele. **a**. Experimental design. **b**. Genotyping of adult fish for intron 2 loxP site. **c**. Results of nested PCR screening for loxP integration into intron 1. **d**. Sequence of intron 1 loxP integration in recovered *tpl145* floxed *tbx20* allele. **e**. Genotyping of “F1” adults. Primer binding sites are shown as black arrows, loxP sites as red or blue triangles, exon as an open box. **f, g**. Induction of tbx20 loss of function by injection of Cre mRNA. **f**. One quarter of embryos obtained by in-crossing *tbx20*^*tpl145*^ heterozygotes and injected with Cre mRNA display a consistent, severe heart development defect. **g**. Genotyping of embryos with severe heart defects (lanes 1–3) and phenotypically normal siblings (lanes 4–8). L, GeneRuler DNA Ladder (ThermoFisher Scientific). Genotyping lanes 1 and 5 correspond to images in **f**. **h, i**. Induction of *tbx20* deletion by 4-hydroxytamoxifen. *tbx20*^*tpl145*^ heterozygote was crossed to Tg(*ubi*:CreERT2) line. GFP-positive embryos were collected at 2dpf and incubated with 5μM 4-HT for 24 hours. **j**. Adults raised from Cre-injected tbx20^tpl145/+^ embryos were incrossed, resulting in approximately 1/4 of embryos (36/128, 28%) with severe heart defects. **k**. Confirmation of Cre-mediated excision of the second intron of *tbx20* by sequence analysis. **l, m**. Analysis of excision efficiency by qPCR. **l**. Excision efficiency was assessed by qPCR in embryos treated with 4-HT at various concentrations at different time points and in two different *ubb*:CreERT2 driver lines. The y-axis indicates un-excised *tpl*^*145*^ normalized to the untreated control. **m**. Images of *tbx20* phenotypes shown after treatment with 4-HT. Individuals 1, 2, and 3 from **l** correspond to images labeled 1, 2, and 3.

To engineer a floxed allele, we incrossed F1 fish heterozygous for the intron 2 loxP site and injected the embryos with *tbx20* sgRNA9 and nCas9n mRNA, followed by injection of the HDR template oligonucleotide targeting intron 1 (Fig 2a). Injected embryos were raised to adulthood and genotyped for the presence of the intron 2 loxP site. Among 61 adults, 28 were heterozygous and 14 were homozygous for the loxP site (Fig 2b). All 42 were outcrossed and screened by nested PCR for integration of the loxP site into intron 1 (Fig 2c). Nine were positive by 5’ end PCR (3’ with regard to the HDR oligonucleotide), and 12 were positive by both 5’ and 3’ end PCRs. In many cases the 3’ end nested PCR fragment was larger than would be predicted, sometimes by a few hundred bases. For six germline transmitters (candidate founders), we extracted DNA from individual embryos and performed flanking PCR to assess germline mosaicism and to confirm complete loxP site integration (S2 Fig). The larger PCR bands observed in two candidate founders yielded poor quality sequences, and these founders were not followed up further. Sequencing the larger PCR bands from the four other founders revealed a high prevalence of insertions and deletions, most remarkably centered around the oligo-T stretch immediately 3’ to loxP integration (corresponding to the 5’ end of the HDR oligonucleotide). Subsequent re-sequencing of the locus revealed that the chromosome containing the loxP site in intron 2 had the longer 10-nucleotide oligo-T, while our HDR template oligonucleotide contained 9. Nonetheless, one of the candidate founders (NP#39) had transmitted integration of a full-length loxP site, albeit with an insertion of an additional 62 nucleotides immediately 3’ to it (Fig 2d). Siblings of genotyped embryos from founder NP#39 were raised to adulthood.

### Validation of the conditional (floxed) allele of *tbx20*

Eight adult fish that were raised from an outcross of candidate founder NP#39 were tail clipped and screened by flanking PCR (Fig 2e). In four of the fish, a larger PCR band corresponding to a floxed allele was observed (red arrows in Fig 2e). Two of the four purified PCR bands were sequenced directly and found to contain identical full-size loxP integrations with a partial target site duplication. For further confirmation, these fragments were cloned into pJet PCR cloning vector and sequenced, and the resulting floxed allele was designated *tbx20*^*tpl145*^ (Fig 2d).

We then proceeded to incross these “F1” fish heterozygous for *tbx20*^*tpl145*^ and to inject some of the embryos with Cre mRNA as described previously [36]. All uninjected embryos were phenotypically normal (n = 52). This observation, together with the fact that *tbx20* deficiency leads to severe cardiac defects [22, 25, 26], enabled us to conclude that the integration of two loxP sites does not significantly impair *tbx20* expression. Among embryos injected with Cre mRNA, approximately 25% displayed severe cardiac defects at 3 dpf in two independent experiments. We genotyped three embryos with severe cardiac defects and five siblings (Fig 2g), corresponding to images in Fig 2f, and found that the three abnormal embryos appeared to have undergone biallelic Cre-mediated excision of the second exon of *tbx20*. This observation was confirmed by sequence analysis of PCR bands (Fig 2k). In contrast, phenotypically wild type embryos were either homozygous wild type or heterozygous for the (excised) floxed allele. Some Cre-injected embryos were raised to adulthood and genotyped. Adult fish heterozygous for the deletion were incrossed, and one quarter of embryos were found to display severe heart defects indistinguishable from that seen in Cre-injected embryos of the previous generation (Fig 2j).

We next tested if Cre-mediated deletion of the second exon of *tbx20* can be achieved in a temporally-regulated manner. We crossed fish heterozygous for the floxed *tbx20*^*tpl145*^ allele to fish expressing the tamoxifen-inducible CreERT2 under the control of the ubiquitin promoter [37]. Obtained embryos were selected for GFP fluorescence (the marker of Tg(*ubb*:*CreERT2*)), incubated in 4-hydroxytamoxifen for 24 hours between 2 dpf and 3 dpf, and individual embryos were genotyped. Deletion of exon 2 of *tbx20* was readily observed in embryos incubated with 4-hydroxytamoxifen, but not in control embryos (Fig 2h and 2i). As expected, all embryos were phenotypically normal because all had a wild type allele of *tbx20*. Together, these experiments led us to conclude that we have successfully engineered a fully conditional (floxed) allele of *tbx20*.

Many experimental approaches in genomics, proteomics and metabolomics require a large amount of starting tissue. Due to their small size, hundreds to thousands of zebrafish embryos or larvae may be needed for each experiment. Genotyping such large numbers of embryos is impractical, leaving two approaches: morpholino knockdown or germline replacement [38, 39]. We hypothesized that if embryos obtained from incross of parents homozygous for the floxed allele were injected with Cre mRNA, we would be able to obtain clutches of all-mutant embryos. To test this hypothesis, we genotyped adults obtained from *tbx20*^*tpl145*^ incross and identified homozygotes. Homozygous fish were incrossed, and embryos were injected with Cre mRNA at 1-cell stage. As expected, Cre-injected embryos displayed full *tbx20* mutant phenotype, while non-injected embryos were all phenotypically wild type (S3 Fig). Thus, large clutches of all-mutant embryos, along with wild type controls, could be generated using this approach.

### Temporally-regulated loss of *tbx20* function

In zebrafish, *tbx20* is expressed in bilateral cardiac precursors prior to their migration and coalescence. Expression appears to persist through larval development, and is upregulated in response to injury in adult hearts [22, 25–27, 40]. In mouse, cardiomyocyte-specific deletion of *Tbx20* in adults leads to lethal cardiomyopathy and arrhythmia, indicating that TBX20 is required not only for cardiac development, but also for homeostasis [24]. To test when it is required for heart development in zebrafish, we sought to induce ubiquitous loss of *tbx20* function at different time points. While performing the experiments described in the paragraph above, we noted that the *ubb*:*CreERT2* line is multi-copy, e.g. carries multiple unlinked integrations of the transgene. We established two lines, sub-designated *ubb*:*CreERT2*.*C* and *ubb*:*CreERT2*.*F* which were single-copy genetically (50% of progeny positive for the cardiac GFP (*myl7*:eGFP) transgenesis marker [37]), and crossed them a *tbx20*^*tpl122*^ partial deletion mutant obtained during epitope tagging experiments to be described elsewhere (Burg et al., unpublished, and S4 Fig). We then crossed *ubb*:*CreERT2*.*C*, *tbx20*^*tpl122*^ double heterozygotes to *tbx20*^*tpl145*^ floxed homozygotes, and exposed embryos to 4-OHT at different time points. We found that exposure to 0.5 μM 4-OHT at 6 hpf reliably induced a full *tbx20* loss-of-function phenotype. In contrast, only a small subset of embryos exposed to 4-HT at 10 hpf displayed a milder cardiac phenotype, while all embryos exposed at 14 or 24 hpf were phenotypically normal. In order to eliminate the possibility that 4-HT was less effective at 10 hpf compared to 6 hpf, we performed qPCR to assess the completeness of Cre recombination at the DNA level. We found that excision was 99% complete in embryos exposed to 4-HT at 6 hpf, and 94% complete in embryos exposed at 10 hpf (S1 Table). Thus, 4-HT is somewhat less efficient at inducing *ubb*:*CreERT2*-mediated recombination at 6 hpf compare to 10 hpf. Consequently, absence of phenotype in embryos exposed at 10 hpf may not be due to timing loss of Tbx20 function, but instead due to slightly lower efficiency of recombination.

To explore the possibility that 4-HT is taken up less well by older embryos, we next compared the toxicity of 4-HT at 6 vs 10 hpf (S2 Table). We treated pools of embryos from the same clutch with 4-HT ranging in concentration from 5 μM to 60 μM and found that embryos treated with 25 μM 4-HT at 6 hpf had a 73% survival rate, while embryos given the same dose given at 10 hpf had a 100% survival rate. Survival rate at higher doses dropped off significantly faster in embryos treated at 6 hpf compared to 10 hpf; however, even the 10 hpf embryos displayed toxicity effects at doses above 25 μM.

We then repeated our loss-of-function experiment by exposing 10 hpf embryos to 20 μM 4-HT, and 6 hpf embryos to either 5 μM or 0.3 μM 4-HT (Fig 2l, S1 Table), using two different single-copy *ubb*:*CreERT2* drivers. We found the Cre-mediated loss of function was at least 95% efficient in embryos exposed to 20 μM 4-HT at 10 hpf, but the embryos displayed very mild, if any, cardiac abnormalities. Exposure to very low doses of 4-HT at 6 hpf induced near-complete excision of the second exon when *ubb*:*CreERT2*.*C* driver was used. In contrast, excision was significantly less efficient with *ubb*:*CreERT2*.*F* driver. However, we observed that 90%-efficient excision of the second exon was sufficient to induce a severe cardiac defect (Fig 2l and 2m, S1 Table).

We also performed a semi-quantitative test for the time lag of CreERT2-mediated recombination after exposure to 4-HT. Pools of 20 embryos exposed to 5 μM 4-HT at 6 hpf and 10 hpf were collected 30 minutes, 1 hr, 2 hrs and 4 hours after exposure. The excision band was readily detectable after 2 hours in 6 hpf treated embryos, and 1 hour after 4-HT exposure in 10 hpf embryos (S5 Fig), indicating that a significant subset of cells may have lost *tbx20* function by then. Increased intensity of the excision band at 4 hours after exposure indicates that recombination is likely not complete yet at 2 hours after addition of 4-HT.

### Conversion of Cre-revertible Gene Breaking Transposon allele to a floxed conditional allele

Gene Breaking Transposons (GBTs) have been used to generate a large collection of gene trap mutants in zebrafish [36, 41] [42, 43] In a typical scenario, a GBT integrates into an intron of a gene and terminates the expression of the mutated gene, leading to complete loss of function. In most widely used GBT’s, the gene trap cassette is flanked by direct loxP sites. Expression of Cre recombinase leads to very efficient excision of the gene trap cassette, reverting the mutant phenotype and leaving a single loxP site flanked by terminal repeats of the Tol2 transposon. Thus, the addition of a single loxP site should convert such reverted gene traps to fully conditional (floxed) alleles.

**Fig 3:**
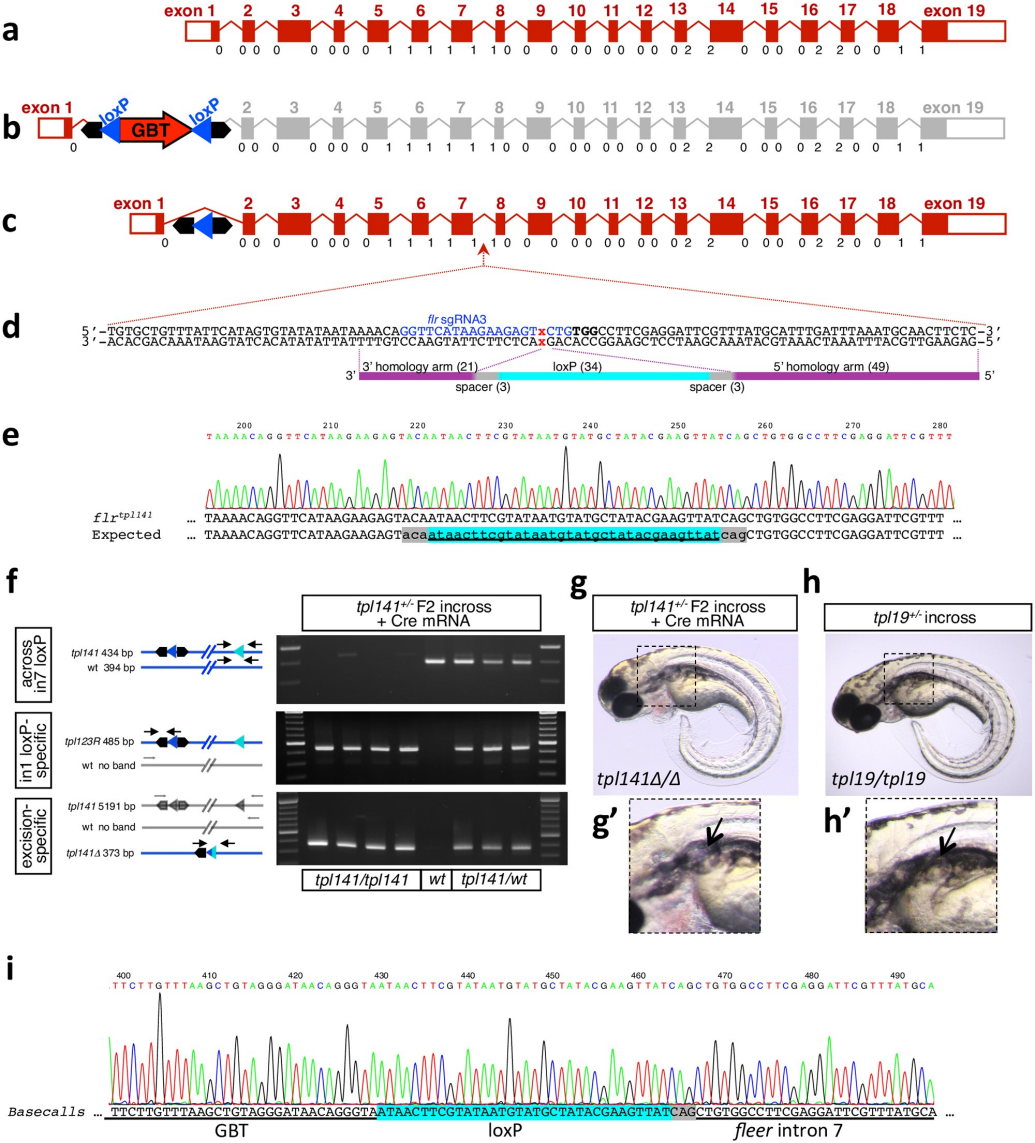
Conversion of a Cre-revertible Gene Breaking Transposon (GBT) allele to a conditional allele. **a**. Diagram of the *fleer* locus. Exons drawn to scale, introns not to scale. Below each intron-exon junction reading frame phase is indicated. **b**. Diagram of the *fleer* gene trap allele *flr*^*tpl19*^. **c**. Diagram *flr*^*tpl19R*^ locus reverted by Cre-mediated excision of the gene trap cassette. **d**. Diagram of *flr* sgRNA3 target site and antisense oligonucleotide HDR template. **e**. Sequence of loxP integration into intron 7, resulting in floxed fleer allele *tpl141*. **f, g, h**. Cre-mediated excision of exons 2–7 of *fleer*. **f**. Induction of *fleer* deletion by Cre mRNA. **g**. One quarter of embryos obtained by in-crossing *fleer*^*tpl141*^ heterozygotes and injected with Cre mRNA display a phenotype consistent with *fleer*^*tpl19*^ homozygotes in **h**, including formation of kidney cysts shown in **g’** and **h’. i**. Sequence of the excision amplicon.

In the *fleer*^*tpl19*^ mutants selected for these experiments, the gene breaking transposon was integrated into the first intron of *fleer*, eliminating expression of downstream exons [36] (Fig 3b). We first injected embryos with Cre mRNA and established a reverted line *fleer*^*tpl19****R***^ (Fig 3c), which was homozygous viable. To engineer a conditional allele, we reviewed the exons of fleer for reading frame phase and the presence of conserved protein domains. Exons 2, 3, and 4 all begin and end in the same reading frame, and mRNAs lacking one or more of them may be translated into functional protein. Furthermore, the first 12 exons all begin and end in either phase 0 or phase 1 (Fig 3a). We therefore decided to attempt to generate a deletion removing all three N-terminal tetratricopeptide repeats (likely important for protein-protein interactions) of *fleer* by engineering a loxP site into intron 7 (Fig 3c and 3d). We sequenced the seventh intron from fish homozygous for the reverted gene trap, designed sgRNAs targeting it and identified *flr* sgRNA3 as being sufficiently active by Surveyor assay as described in (15). To integrate a loxP site, we designed a 110-base HDR template oligonucleotide with asymmetric homology arms, antisense to the PAM. Embryos were injected with *flr* sgRNA3, nCas9n mRNA, followed by injection of the HDR template oligonucleotide. Fish were raised to adulthood and screened for germline transmission of the loxP site by nested PCR. After screening 14 F0 fish, we identified one that transmitted a perfect integration of the loxP site. Siblings were raised to adulthood, and one out of twenty-one adult F1 fish was positive for the loxP integration (sequenced in Fig 3e) and was assigned allele name *flr*^*tpl141*^.

We then proceeded to test the conditionality of this allele by incrossing *flr*^*tpl141*^ F2 heterozygous fish and injecting embryos with Cre mRNA (Fig 3f). Twenty-five percent of the progeny were expected to be homozygous for the floxed allele, and approximately one quarter of the injected embryos displayed a phenotype consistent with previously published *flr*^*m477*^ chemical mutant, as well as *flr*^*tpl19*^ gene trap mutant [29, 36]. Embryos were first genotyped using primers spanning the intron 7 loxP site, which amplified the WT allele as well as any non-excised *flr*^*tpl141*^. The same embryos were also genotyped for the loxP site in intron 1, and amplicons were detected in embryos carrying *flr*^*tpl141*^. Finally, embryos were genotyped by PCR across both loxP sites, with the excision product being readily detected in all *flr*^*tpl141*^-positive embryos embryos injected with Cre mRNA (Fig 3f, bottom panel). In this last experiment, any non-excised floxed allele was undetectable due to large amplicon size. All genotyped embryos with the *fleer* mutant phenotype were homozygous for *flr*^*tpl141*^, while all heterozygous and WT siblings were phenotypically wild-type.

### *aldh1a2* is the only retinal dehydrogenase highly expressed in regenerating zebrafish hearts

Pharmacological perturbation of retinoic acid (RA) signaling leads to severe defects in regeneration across various model systems and tissues (reviewed in [44]). Conditional mutants capable of inactivating RA signaling in specific cell types are needed to better understand the requirement of RA signaling for regeneration. Zebrafish have at least three genes coding for retinal dehydrogenases: *aldh1a2*, *aldh1a3* and *aldh8a1*. Upregulation of *aldh1a2* expression serves as the hallmark of endocardial and epicardial activation, making it an attractive target for conditional mutagenesis. In order to test which retinal dehydrogenases are likely to make an important contribution to retinoic acid production during regeneration, we analyzed RNA sequencing data from zebrafish hearts at 1, 3 and 5 days after cryoinjury and confirmed that *aldh1a2* is highly expressed and upregulated in response to cryoinjury [45]. In contrast, *aldha8a1* was expressed at a much lower level, and expression of *aldh1a3* was undetectable (S6 Fig). The observation that other RA-synthesizing enzymes are poorly, if at all, expressed in the regenerating heart suggests that loss of *aldh1a2* function is likely to lead to severe reduction in RA signaling in the regenerating heart. This hypothesis cannot currently be directly tested, as *aldh1a2* mutants display pleiotropic embryonic phenotypes and do not survive [30, 31].

### Deletion of exon 8 of *aldh1a2* results in a null mutant

**Fig 4:**
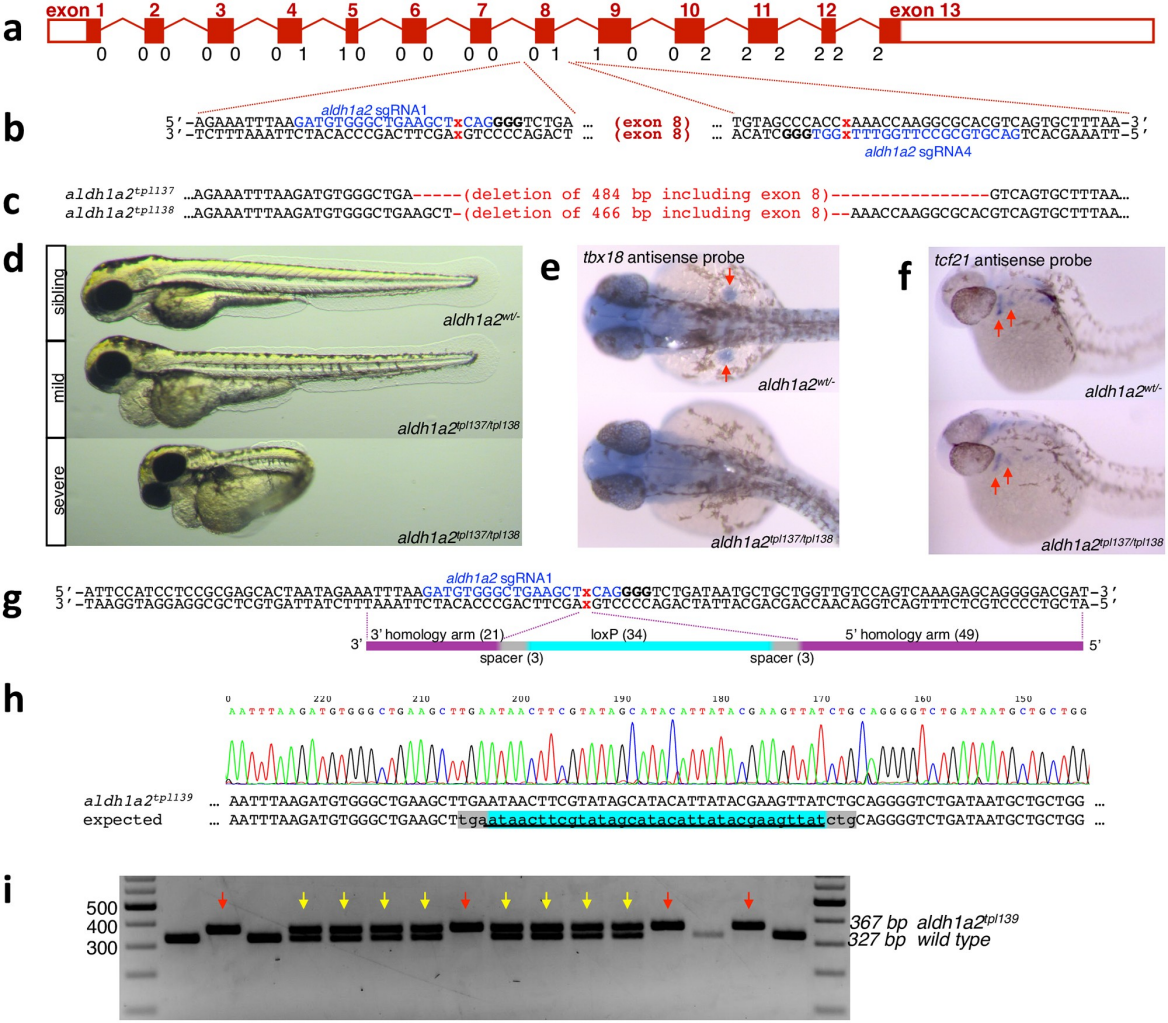
Mutagenesis of *aldh1a2*. **a**. Diagram of the *aldh1a2* locus. Exons drawn to scale, introns not to scale. Below each intron-exon junction reading frame phase is indicated. **b**. Two highly active intronic *aldh1a2* sgRNAs, *aldh1a2* sgRNA1 and *aldh1a2* sgRNA4, flank exon 8. Sequence corresponding to single guide RNA is shown in blue, PAM motif is in bold, and expected Cas9 cut site is indicated by a red x. **c**. Two independent *aldh1a2* exon 8 deletion alleles recovered after co-injection of *aldh1a2* sgRNA1 and *aldh1a2* sgRNA4 along with nCas9n mRNA. For Sanger sequencing of the alleles, see S3 Fig. **d, e**. Deletion of exon 8 of *aldh1a2* results in expected loss-of-function phenotype. F1 fish heterozygous for *aldh1a2*^*tpl137*^ and *aldh1a2*^*tpl138*^ deletions were crossed to each other. **d**. Images of 3 dpf embryos displaying wild type (top) and the expected *aldh1a2* loss of function phenotypes: lack of pectoral fins, shortened hindbrain brain and cardiac edema. In addition, most of the 3 dpf embryos displaying these phenotypes had curved tails (d, bottom) consistent with uneven left/right somite numbers. **e**. *aldh1a2*^*tpl137/tpl138*^ trans-heterozygotes lack pectoral fin buds as revealed by loss of *tbx18* expression at 32 hpf. **f**. Expression of *tcf21* persists in the first and second branchial arches of *aldh1a2*^*tpl137/tpl138*^ trans-heterozygotes at 32 hpf. **g**. Diagram of the HDR template oligonucleotide used to knock in the loxP into *aldh1a2* sgRNA1 target site. **h**. Sequencing of the precise loxP knockin allele. Additional knock-in alleles recovered from other F0 families are shown in S5 Fig. **i**. Genotyping of 16 phenotypically normal 3 dpf embryos from a cross between F1 fish heterozygous for *aldh1a2*^*tpl139*^ loxP knock-in results in Mendelian ratios of *aldh1a2*^*tpl139/tpl139*^ (red arrows), *aldh1a2*^*tpl139/wt*^ (yellow arrows) and *aldh1a2*^*wt/wt*^ embryos. PCR fragments from two of the embryos were sequenced to further confirm homozygosity for the loxP site.

For *aldh1a2*, we decided to target exon 8 because it encodes a part of the highly conserved alcohol dehydrogenase domain (Fig 4). In addition, it begins and ends in different phases of the reading frame. With exon 8 deleted, in-frame transcripts can be generated by either skipping exons 5–7 or by skipping exon 9. Both of these scenarios would remove additional parts of the dehydrogenase domain with highly conserved amino acids. We sequenced the region flanking exon 8 from four wild type TLF fish (two males and two females), and designed four sgRNAs (S7 Fig). We tested their activity by direct sequencing of PCR fragments generated on pools of injected embryos [46] and found that *aldh1a2* sgRNA1 and *aldh1a2* sgRNA4 were highly active, while *aldh1a2* sgRNA2 and *aldh1a2* sgRNA3 produced very few indels.

To test if deletion of the eighth exon would lead to loss of function phenotypes comparable to those of previously published *aldh1a2* mutants, we co-injected both *aldh1a2* sgRNA1 and *aldh1a2* sgRNA4 along with nCas9n mRNA, and observed the appearance of a PCR band corresponding to the expected deletion product. Injected embryos were raised to adulthood, and two incrosses of F0 fish were performed. In pools of embryos from both incrosses, a PCR band corresponding to the expected deletion size was readily observed (S7 Fig). Sibling F1s were raised to adulthood and genotyped. Two out of seventeen analyzed F1 fish from incross A were found to be heterozygous for a deletion of 484 base pairs (Fig 4c, S7 Fig). One out of fifteen analyzed F1 fish from incross B was found to be heterozygous for a deletion of 466 base pairs (Fig 4c, S7 Fig). We crossed the F1 fish heterozygous for *aldh1a2*^*tpl137*^ allele to the F1 fish heterozygous for the *aldh1a2*^*tpl138*^ allele. Approximately 25 percent of 3 dpf embryos lacked pectoral fins and had moderate to severe pericardial edema. In addition, the majority of embryos displaying these phenotypes also displayed curved tail consistent uncoordinated left-right somitogenesis as previously reported [47] (Fig 4d). The absence of pectoral fin buds at 30 hpf was confirmed by in situ hybridization using a probe against *tbx18*. Since both *tcf21* and *aldh1a2* are expressed in the branchial arches and both mutants display a defect in the development of branchial arch-derived structures, we also performed *in situ* hybridization using a probe against *tcf21*. We found that while *tcf21* expression in the posterior branchial arches is absent in *aldh1a2* mutants, expression of *tcf21* in the first and second branchial arches of *aldh1a2* mutants persists (Fig 4f). We therefore concluded that *aldh1a2* is not required for *tcf21* expression in the first and second branchial arches. Linkage of these phenotypes to *aldh1a2* exon 8 deletion was confirmed by 3-primer genotyping PCR (S8 Fig).

### Integration of loxP site into intron 7 of *aldh1a2*

We designed a 110 base long HDR template oligonucleotide (Fig 4g) and injected it into embryos which were also injected with *aldh1a2* sgRNA1 and nCas9n mRNA. Integration of the loxP site was readily detected in pooled DNA of injected embryos as described in Materials and Methods. Embryos were raised to adulthood and seven incrosses (14 F0 fish) were screened for germline transmission of integration of the loxP site by nested PCR. Five pairs were positive by nested 5’ and 3’ PCR. In three cases, loxP integrations transmitted through the germline also contained an insertion of additional sequences within the 5’ homology arm (S9 Fig). One founder pair (R9x10) transmitted a precise integration of the loxP site, and 3/8 individual embryos analyzed were found to be heterozygous for the loxP-containing allele. Founder pair R13x14 transmitted integration of the loxP site with a single nucleotide substitution within the 5’ homology arm. Adult F1s raised from these crosses were genotyped by PCR (S9 Fig). Three out of fourteen adults from pair R9x10 proved to be heterozygous for an allele containing a precise integration of the loxP site, designated *aldh1a2*^*tpl139*^ (Fig 4h). One out of twelve adults from the F1 family R13x14 was heterozygous for the loxP integration with a single nucleotide substitution, and we established a backup loxP-containing allele, designated *aldh1a2*^*tpl140*^. Two F1 fish heterozygous for *aldh1a2*^*tpl139*^ were incrossed, and all embryos appeared phenotypically normal at 5 days post fertilization. Sixteen were genotyped for the presence of the loxP site, and four were found to be homozygous (Fig 4i). We concluded that integration of the loxP site into intron 7 does not significantly impair expression of *aldh1a2*.

### Integration of loxP site into the 5’ UTR of *tcf21*

In contrast to the first four large, multiple exon genes that we targeted, *tcf21* is a small, two-exon gene, making it impossible to integrate both loxP sites into introns. We speculated that the 5’ UTR might be a suitable target for loxP integration. First, the loxP site does not contain ATG codons that would cause premature translation initiation. Second, most regulatory transcription factor binding sites are expected to be upstream of the transcription initiation site (not immediately downstream), making it less likely that the integration of a loxP site would disrupt a transcription factor binding site. Finally, 3’ UTRs may contain regulatory elements such as microRNA binding sites which are rather difficult to predict. We designed two short guides targeting the 5’ UTR of *tcf21*, and both were reasonably efficient at inducing DSBs (S10 Fig). We decided to first perform loxP integration into the *tcf21* sgRNA5 target site and designed a HDR repair oligonucleotide with asymmetric arms (S11 Fig). Embryos injected with *tcf21* sgRNA5, nCas9n mRNA and the HDR oligonucleotide were raised to adulthood and screened for germline transmission of the loxP site. Out of nine incrosses, three were positive for germline transmission of a loxP site by nested PCR. One had an insertion of approximately 100 nucleotides and was not analyzed further. Single embryos from the other two transmitter pairs were analyzed. An incomplete loxP site was identified in one of the pairs, and integration of a full loxP site without indels was found in the second pair (*tcf*inxB). Siblings of analyzed embryos from *tcf*inxB were raised to adulthood and genotyped. Of twenty-four siblings that were genotyped, four were positive by PCR for integration of the loxP site. All four were sequenced and were found to have the same precise integration, designated *tcf21*^*tpl144*^ (S11 Fig). To test for the possibility that integration of loxP site impairs expression of *tcf21*, we crossed *tcf21*^*tpl144*^ F1 fish to a previously established *tcf21* frameshift mutant *tcf21*^*tp119*^ [15]. All embryos were phenotypically normal, demonstrating that the loxP-containing allele *tcf21*^*tpl144*^ is functionally wild type.

## Discussion

**Fig 5:**
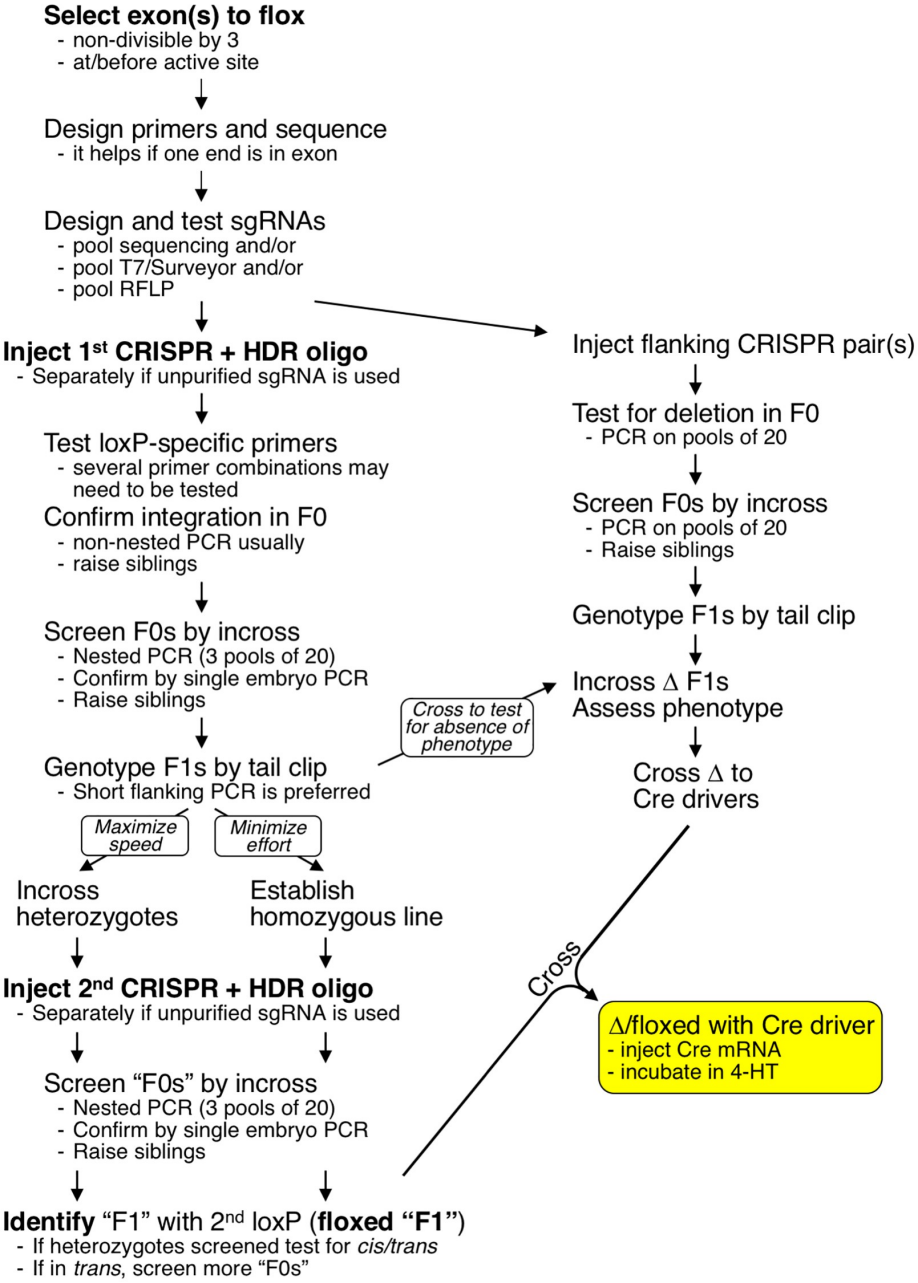
Conditional mutagenesis pipeline. Upon deciding which exon to flox, we recommend sequencing the target sites to identify polymorphisms compared to reference genome. Next, sgRNAs should be designed and tested by either direct sequencing of PCR fragments, T7 endonuclease assay or loss of a restriction enzyme site on bulk DNA from pooled embryos. Once active sgRNAs have been identified, experiments integrating the first loxP site should be performed. In the absence of conclusive data that certain HDR template performs significantly better than others (such experiments are not practical at the only level that matters–germline transmission), we recommend using the design we successfully used to integrate loxP into *fleer*, *aldh1a2* and *tcf21*: antisense to PAM, with 49-base 5’ homology arm and 21-base 3’ homology arm, with 3-nucleotide spacers flanking loxP site. As injected embryos are being raised, we then recommend to optimize nested PCR screening conditions DNA from pools of injected embryos. We found “plain” Taq polymerases (NEB #M0270, Thermofisher Scientific 2x PCR Master Mix Cat# AB-0575/DC and #EP0402, or similar) to be most suitable for nested PCR. In contrast, high-performance mixes such as Platinum Taq (Thermofisher #10966026) or Kapa 2G Fast ReadyMix + dye (Kapa Biosystems- KM5101) yield very high background and may only be used for the second (nested) reaction. It is also very helpful if primers for one end of the nested PCR are anchored within an exon. We recommend generating a deletion allele in parallel with integration of the first loxP site. Once highly active sgRNAs are identified, we recommend injecting a pair of sgRNAs flanking the exon to be floxed in order to confirm that removal of selected exon will yield an overt phenotype. We have been able to very efficiently delete exon 8 of *aldh1a2* using sgRNAs spaced just over 450 base pairs, but larger deletions are certainly feasible too (1, 2). An additional benefit of a deletion allele is that it can be crossed to Cre drivers of interest, eliminating the need to back-cross floxed allele to obtain homozygotes. Screening for germline transmission should be performed by nested PCR on pools of embryos obtained from incross. Positive crosses should be analyzed by performing short flanking PCR (ideally under 400 base pairs) on DNA from individual embryos. Bands corresponding to loxP-containing allele should be extracted from gel and sequenced to ensure presence of intact loxP site. Siblings of screened embryos should be raised to adulthood and loxP-positive F1s should be identified by flanking PCR as well. Two strategies can be used for integration of the second loxP site. If speed is the main priority, loxP-positive F1s can be in-crossed and second sgRNA/HDR oligonucleotide can be injected. The main drawback of this strategy that there is only 50% likelihood that the second loxP site will integrate into a chromosome already containing the first loxP. It is therefore necessary to genotype adults for presence of the first loxP site before out-crossing. Even though we successfully used this strategy to engineer a floxed allele of *tbx20*, we consider it impractical and would generally recommend to first generate adults homozygous for the first loxP site, incross them and then inject the second sgRNA/HDR oligonucleotide.

We have used oligonucleotide-mediated homology directed repair to integrate loxP sites into five different locations in the zebrafish genome: intron 1 of *tbx20*, intron 2 of *tbx20*, intron 7 of *fleer*, intron 7 of *aldh1a2*, and the 5’ UTR of *tcf21*. Across all five loci, we have observed remarkably high rates germline transmission and germline mosaicism: the median rate of germline transmission of a complete loxP site was 6.3% (1 out of 16 F0 fish screened), while the median rate of germline mosaicism was 20% (1 out of 5 F1 fish screened from germline-transmitting founders) (S3 Table). A conditional mutagenesis workflow based on our experience is provided in Fig 5.

Our data clearly demonstrates the feasibility of conditional mutagenesis by oligonucleotide-mediated sequential integration of loxP sites into the zebrafish genome. Two aspects of our method are likely to have contributed to its success. First, based on the previous observation of a high rate of indels at the junctions between the integrated sequences and homology arms [15], we flanked the loxP site by 3-nucleotide spacer sequences. A loxP site consists of an 8-base core flanked by 13-base inverted repeats. The melting temperature of the inverted repeats is calculated at 32°C, meaning that a loxP site may form a hairpin in zebrafish embryos. The 3-nucleotide spacers may have facilitated homology-directed repair by providing spacing between the hairpin and homology arms. Indeed, successful oligonucleotide-mediated integration of the mlox site, which has 3 substitutions in one of the inverted repeats, has been reported by two laboratories [20, 21]. We have not directly compared the efficacy of oligonucleotide templates with short or long homology arms, but 110-base long oligonucleotides with a longer 5’ arm, antisense to the PAM, have been successfully used in the majority of our experiments, leading us to recommend this particular design. Further gains in HDR efficiency may be achieved by using phosphorothioate-modified oligonucleotides [48, 49]. The second aspect of our method is screening for germline transmission by nested PCR. Over the course of our work, we have used several different loxP-specific primers and find that even in nested PCR, different loxP-specific primers need to be tested with different genomic primers to identify optimal pairs. The palindromic nature of the loxP site likely contributes to poor performance of loxP-specific primers.

Given that it takes a considerable amount of time to engineer a conditional mutant, it makes sense to ensure that the mutant will display a phenotype as early as possible. This is a two-step process. To determine the likelihood that the selected gene will be required for biological function of interest, it may be important to examine the expression of homologs which may provide identical or similar biochemical activity, as we have done for retinal dehydrogenases in regenerating hearts. Second, it is important to ensure that loss of the exon selected for floxing will lead to a severe or null phenotype. Using *aldh1a2* as the model locus, we demonstrate that a pair of sgRNAs can be used to direct very efficient excision of the target exon. While our sgRNA target sites were less than 500 base pairs apart, larger deletions can also be readily induced using sgRNAs spaced over 15 kilobases apart [50]. An additional benefit of a deletion allele is that it can be crossed to Cre drivers of interest, eliminating the need to back-cross the floxed allele to obtain homozygotes (Fig 5).

An alternative way to ensure that a conditional mutant will display a phenotype of interest is to start from a Cre-revertible Gene Breaking Transposon allele [36, 41–43]. The very high efficiency of Cre/lox recombination makes it possible to establish a line homozygous for Cre-reverted GBT allele with minimal effort. Only one loxP site then needs to be integrated to convert such Cre-reverted gene traps into fully conditional alleles, as demonstrated with *fleer*.

One of the challenges of using CRISPR/Cas9 to integrate loxP sites into the introns is that intronic sequences are often of low complexity, have low G/C content, and are repetitive. These features may limit targeting possibilities by making it difficult to design highly efficient sgRNAs. Requirement for GC-rich 5’-NGG-3’ PAM sequence poses an additional complication. Nonetheless, we were able to find suitable targets in each gene and intron of interest. We have used several methods to assess guide RNA activity: T7 endonuclease assay, loss of restriction enzyme site, and direct sequencing of PCR fragments [15, 46]. Activity assessment is always performed on DNA extracted from a pool of at least 10 embryos, thus providing a quantitative estimate of average editing efficiency. In our experience, about half of the guides meet the minimum requirement of at least 50% editing efficiency. CRISPR/Cas9 systems recognizing A/T-rich PAMs, such as Cpf1 [51, 52], will make editing of intronic sites even more straightforward.

Conditional mutagenesis relies on the availability of high quality, tissue-specific drivers of tamoxifen-inducible Cre recombinase. In the mouse, the majority of drivers have been engineered by integrating CreERT2 into the specific loci, thus likely fully recapitulating the expression of the “driver” gene. In zebrafish, drivers are typically engineered by Tol2-mediated transgenesis of an expression cassette. High activity of Tol2 transposase [53–55] means that many lines, at least initially, are multicopy, with each copy subject to position effects (for extreme examples, see [56–58]). As we have shown for two different copies of *ubb*:CreERT2, it is not sufficient to validate a given transgenic construct; the specific transgene integration, as a single-copy transgenic line, has to be validated for use in loss-of-function experiments.

Our genome editing experiments were performed in three different genetic backgrounds: AB (*tbx20*), TLF (*aldh1a2* and *tcf21*), and undefined pet store-derived (*fleer*). The majority of our experiments were performed in the United States, but initial integrations of loxP sites into *tbx20* were recovered in Germany. We therefore believe that our conditional mutagenesis methodology can be readily adapted by any laboratory and performed in any genetic background. Additionally, the oligonucleotide templates we used for homology directed repair are both inexpensive and widely available. The ability to efficiently generate conditional mutants in zebrafish offers new possibilities for understanding the roles of pleiotropic genes, and essential for studies of post-embryonic processes such as regeneration.

## Materials and methods

### Ethics statement

All animal experiments described in this manuscript have been approved by Temple University IACUC committee under protocol numbers ACUP 4354, ACUP 4709 and ACUP 4164.

### nCas9n mRNA synthesis

nCas9n was prepared as previously described [15]. pT3TS-nCas9n [59] was linearized with XbaI and transcribed using the T3 mMessage mMachine *in vitro* transcription kit (ThermoFisher Scientific AM1348). Transcribed mRNA was purified using the Qiagen RNeasy MinElute kit (74204), diluted to 150 ng/μL in RNAse free water (ThermoFisher Scientific AM9937) and 2 μL aliquots were made. Aliquots were stored at -80°C.

### sgRNA synthesis

sgRNA was prepared as previously described [15]. We used cloning-free PCR method similar to the ones described previously [60, 61] to produce sgRNA synthesis template. We first performed PCR using guide-specific and M13F primers on DR274 [62] template. Guide-specific and all other primers used in this study are listed in S4 Table. We then performed agarose gel electrophoresis and purified the bands using GeneJet Gel Extraction kit (ThermoFisher Scientific K0692). Purified band was used as the template for the second PCR reaction with sgT7 and sgRNA-R primers. The obtained PCR fragment was purified using GeneJet PCR purification kit (ThermoFisher Scientific K0702) and used as the template for *in vitro* transcription using MEGAshortScript T7 transcription kit (AM1354). After RNA synthesis, concentration of sgRNA was assessed by agarose gel electrophoresis. Concentration was estimated by comparing to RiboRuler RNA ladder (SM1833). Unpurified transcription reaction mix was diluted to approximately 60 ng/μL and 8 μL aliquots were made. Aliquots were stored at -80°C.

### Microinjection

An 8 μL aliquot of sgRNA was thawed and mixed with 2 μL aliquot of nCas9n mRNA. 3 nL of mix were injected into the yolks of 1-cell zebrafish embryos as described previously [15, 63].

Appropriate loxP HDR oligonucleotide was diluted to 50 ug/μL in RNAse free water, and 1 nL was injected into the yolks of zebrafish embryos immediately after the RNA injection.

### Testing CRISPR activity in injected embryos

At 3–5 dpf, 20 embryos were pooled into a standard microcentrifuge tube and lysed. 0.8–1.0 μL of lysate was used as the template for PCR reactions in 20–25 μL volume. All PCR reactions were performed using either NEB 2X Taq Master Mix (M0270L), Thermo Scientific Taq (EP0402 and 2x PCR Master Mix Cat# AB-0575/DC), or Kapa 2G Fast ReadyMix +dye (Kapa Biosystems-KM5101). CRISPR activity was assessed by either direct sequencing of PCR amplicons, T7 assay (NEB M0302S), Surveyor assay (IDT 706025), or RFLP.

To test for loxP integration, PCR reactions were performed using different combinations of flanking and loxP-specific primers, either on pools of 10–20 injected embryos or on 8 individual embryos.

### Screening for germline transmission of loxP integrations by nested PCR

Embryos from F0 incross or outcross were pooled into batches of 20 and DNA prepped. Three batches from each pair were tested for loxP integrations by nested PCR (gene specific primers described below). The first flanking PCR was run and amplification was confirmed by gel electrophoresis. The PCR was purified, diluted 1:100 with water, and 1 μL was used as a template for the 5’ and 3’ nested reactions. Siblings from positive crosses were raised to adulthood. All PCR reactions were performed using either NEB 2X Taq Master Mix, Thermo Scientific Taq or Kapa 2G Fast ReadyMix. DNA ladders used were Thermo Scientific GeneRuler DNA ladder (SM0331) and ThermoFisher Scientific 100 bp DNA ladder (#SM0241)(Fig 1d).

### Screening F1s for loxP integrations by short flanking PCR

Adult F1 fish were tail clipped, DNA prepped, and genotyped by running a short flanking PCR (gene specific primers described below) designed to amplify approximately 450 bp or less. PCRs were run on 2% agarose gels, and amplicons of appropriate size were purified and sequenced to confirm loxP integration. All PCR reactions were performed using either NEB 2X Taq Master Mix, Thermofischer Scientific Taq or Kapa 2G Fast ReadyMix. DNA ladders used were Thermo Scientific GeneRuler DNA ladder.

### Reversion of the *fleer* gene trap

Fish containing the *fleer* GBT were incrossed and embryos were injected with 25 pg Cre mRNA. After being raised to adulthood, the injected fish were tail clipped and genotyped for the reverted gene trap using the flrUTR-F1/Tol2-F8 primer pair.

### Cre mediated excision of floxed exons

*tbx20*^*tpl145*^: Embryos from the incross of *tbx20*^*tpl145*^ heterozygotes were injected with 25 pg Cre mRNA. Individual embryos were genotyped by PCR using the tbx20In1-F4/tbx20In2-R2 primer pair.

*fleer*^*tpl141*^: Embryos from the outcross of the heterozygous floxed *fleer*^*tpl141*^ to wt were injected with 25 pg Cre mRNA. Individual embryos were genotyped by PCR for the presence of the intron 7 loxP site using the flrEx7-F1/flrIn7-R4 primer pair, for the presence of the intron 1 loxP site using the flrUTR-F1/loxP-R1 primer pair, and for the excision product using the tol2-R7/flrIn7-R4 primer pair.

### Tamoxifen inducible CreERT2 mediated excision of floxed exons

Fish heterozygous for either floxed *tbx20*^*tpl145*^ or *fleer*^*tpl141*^ were crossed to the transgenic line Tg(ubi:CreERT2). Embryos were screened for GFP (transgene marker) at 2dpf, and GFP positive embryos were split and incubated with either 5 μM 4-HT or ethanol (control) for 24 hours. Individual embryos were DNA prepped and genotyped using the primer pair tbx20In1-F4/tbx20In2-R1 for *tbx20*, or as described for Cre injection for *fleer*.

### Expression of RA-synthesizing enzymes in the regenerating zebrafish heart

Expression values of RA-synthesizing genes (*aldh1a2 and aldh8a1*) in zebrafish hearts at various time points post sham operation or cardiac injury were extracted from the whole-genome RNAseq datasets previously published [45] Fragments per kilobase of transcript per million mapped reads (FPKM values) were summarized and presented in S3 Fig.

### Mutagenesis of *aldh1a2*

*aldh1a2*sgRNA1 and *aldh1a2*sgRNA4 were co-injected along with nCas9n mRNA as previously described. Injected fish were raised to adulthood, crossed, and screened for germline transmission of deletion alleles by PCR using the aldh1a2-F2/aldh1a2-R6 primer pair. Siblings were raised to adulthood, tail-clipped, and screened for the presence of the deletion by 3 primer PCR using the primers aldh1a2-F4/aldh1a2-R1/aldh1a2-R6.

### Whole mount in situ hybridization

In situ hybridization was performed on embryos obtained from the incross of *aldh1a2*^*tpl137*^ and *aldh1a2*^*tpl138*^ heterozygotes as described [64]. *tcf21* cDNA was amplified using tcf21-5′ UTR-F1 and tcf21-3′ UTR-R2. *tbx18* cDNA was amplified using tbx18-5′ UTR-F1 and tbx18-R10. Each fragment was cloned into pGEM-T vector for transcription using the Ambion T7 Megascript kit (AM1334) and labeling using Roche-DIG labeling mix (Roche 11277073910).

### *tbx20* qPCR

Fish homozygous for the floxed *tbx20*^*tpl145*^ allele were crossed to fish heterozygous for tg(*ubb*:*CreERT2*) and the *tbx20* deletion allele, *tbx20*^*tpl122*^. Embryos were treated with the stated concentrations of 4-HT or ethanol at 6 or 10 hpf for 24 hours. Embryos were scored for phenotype, lysed at 3dpf, and genotyped using the primer pair tbx20In1-F1/tbx20In2-R2. All embryos used for qPCR were genotyped for *tbx20*^*tpl145/tpl122*^ or *tbx20*^*tpl145Δ/tpl122*^. qPCR was performed using a Roche LightCycler 480 and LightCycler 480 SYBR Green I Master Mix (04707516001). Primer pairs used for qPCR were tbx20In1-F1/tbx20In2-R2 and aldh1a2-F5/aldh1a2-R7. Mean Cp values for tbx20 were normalized to aldh1a2 in Microsoft Excel.

### Gene-specific primer pairs

#### *tbx20* intron 1

Guide/HDR oligo: *tbx20*sgRNA9/ tbx20-sg9-loxP-F1
Flanking PCR: tbx20In1-F5/tbx20ex2-R6 or tbx20In1-F1/tbx20In2-R1
5’ nested: tbx20In1-F4/loxP-U1 or tbx20In1-F1/lox-U1
3’ nested: tbx20Ex2-R4/loxP-U2 or tbx20In2-R2/loxP-R1
Short flanking PCR: tbx20In1-F2/tbx20Ex2-R1 or tbx20In1-F4/tbx20Ex2-R1

#### *tbx20* intron 2

Guide/HDR oligo: *tbx20*sgRNA10/ tbx20-sg10-loxP-F1
Flanking PCR: tbx20In1-F1/tbx20In2-R1
5’ nested: tbx20In1-F1/lox-U1
3’ nested: tbx20In2-R2/loxP-R1
Short flanking PCR: tbx20Ex2-F1/tbx20In2-R2

#### *fleer* intron 7

Guide/HDR oligo: *flr*sgRNA3/ flrup3-HDR-L
Flanking PCR: flrEx7-F1/flrIn7-R1
5’ nested: flrEx7-F2/loxP-R1
3’ nested: flrIn7-R2/loxP-F1
Short flanking PCR: flrEx7-F1/flrIn7-R4

#### *aldh1a2* intron 7

Guide/HDR oligo: *aldh1a2*sgRNA1/aldh1a2sg1-loxP-HDR-L
Flanking PCR: aldh1a2-F1/aldh1a2-R6
5’ nested: aldh1a2-F2/loxP-U1
3’ nested: aldh1a2-R1/loxP-U2
Short flanking PCR: aldh1a2-F4/aldh1a2-R1

#### *tcf21* 5’ UTR

Guide/HDR oligo: *tcf21*sgRNA5/tcf21sg5-HDR-L
Flanking PCR: tcf21-F9/tcf21-R1
5’ nested: tcf21-F10/loxP-R1
3’ nested: tcf21-R5/loxP-F1
Short flanking PCR: tcf21-F1/tcf21-R5

## Supporting information

S1 Fig Assessment of guide RNA activity by T7 endonuclease assay and by loss of a restriction enzyme site. **a**. Diagram of the tbx20 locus around tbx20sgRNA9 cut site. Underneath, expected fragment sizes after digestion of T7 endonuclease or BspLI restriction endonuclease. **b**. The BspLI restriction enzyme site overlaps the expected double strand break site, indicating that almost all indels will result in loss of BspLI site. **c**. Gel electrophoresis of PCR fragments digested with T7 Endonuclease or BspLI restriction endonuclease.
(PDF)

S2 Fig Screening for integrations of the second loxP site into *tbx20*. **a, b**. Sequencing of individual embryos from outcross of candidate founder #NP29. One out of sixteen embryos contained an incomplete loxP site with a small deletion (**a**), and two out of sixteen embryos were heterozygous for an allele with a complete loxP site containing a single nucleotide substitution, with an insertion of additional 64 nucleotides (**b**). **c**. Sequencing of individual embryos from cross of founder #NP33. Nine out of sixteen individual embryos were heterozygous for this integration of an incomplete loxP site. **d**. Sequencing of individual embryo from cross of founder #NP39. Five out of sixteen individual embryos were heterozygous for integration of a complete loxP site with an insertion of additional 62 nucleotides. Floxed allele *tbx20*^*tpl145*^ (Fig 2) was established from this founder. **e, f**. Sequencing of individual embryos from outcross of founder #NP58. Four out of sixteen individual embryos were heterozygous for an integration of an incomplete loxP site with insertion of additional 21 nucleotides (**e**), and one out of sixteen was heterozygous for integration of complete loxP site containing a single nucleotide substitution, with insertions of 16 and 55 nucleotides on each side of the loxP site (**f**). Note that all large insertions appear to be partial target site duplications.
(PDF)

S3 Fig Generation of “all-mutant” clutches of embryos. **a**. Experimental design. Fish homozygous for the floxed allele are incrossed, and half the embryos are injected with Cre mRNA. **b-e**. Representative images of Cre-injected (**b,d**) and un-injected siblings (**c,e**) at 1 dpf (**b,c**) and 3 dpf (**d,e**).
(PDF)

S4 Fig Sequence of *tbx20tpl122*. *tpl122* contains a 241 bp deletion removing most of exon 2. *tpl122* homozygotes display a consistent and strong *tbx20* phenotype.
(PDF)

S5 Fig Time course of excision after 4-HT exposure. **a**. Embryos were treated with 5 μM 4-HT at either 6 hpf or 10 hpf. Embryos were pooled (n = 20) and collected at 30, 60, 120, and 240 minutes after exposure. 0 indicates a pool of siblings not exposed to 4-HT. Note: 6 hpf and 10 hpf not treated control PCRs were performed on the same DNA sample.
(PDF)

S6 Fig Expression on RA-synthesizing enzymes in response to heart injury. Expression of retinoic acid synthesizing genes (*aldh1a2 and aldh8a1*) in adult zebrafish hearts at various time points post sham operation or cardiac injury in fragments per kilobase of transcript per million mapped reads (FPKM). *aldh1a2* was highly upregulated in response to injury. *aldh1a3* was not detectable at any tested time point. hps, hours post sham injury, hpi, hours post injury, dps, days post sham injury, dpi, days post injury.
(PDF)

S7 Fig Testing of sgRNAs targeting *aldh1a2* and isolation of exon 8 deletion lines. **a**. Sequencing of PCR fragments amplified from bulk DNA obtained from pools of 6–10 embryos injected with *aldh1a2* sgRNA1, *aldh1a2* sgRNA2, *aldh1a2* sgRNA3 and *aldh1a2* sgRNA4 along with nCas9n mRNA. The sequences are in 5’ -> 3’ with regard to *aldh1a2* locus. Sequences corresponding to single guide RNA are shown in blue, PAM motifs are bold, and expected Cas9 cut sites are indicated by red arrows. Direction of the sequencing reaction is shown by black arrows above. **b**. Detection of exon 8 deletions in DNA from pools of 20 embryos from F0 incrosses A (ΔinxA) and B (ΔinxB). Diagram of the locus on top corresponds to gel electrophoresis results on the botttom, with a yellow arrow pointing to the expected deletion band. **c**. PCR genotyping of adult F1 fish. Two out of 17 F1 fish from ΔinxA family carry a deletion, and one out of 15 F1 fish from the ΔinxB family carries a deletion. **d**. Sequencing of *aldh1a2*^*tpl137*^ deletion allele recovered from ΔinxA family. **e**. Sequencing of *aldh1a2*^*tpl138*^ deletion allele recovered from ΔinxB family.
(PDF)

S8 Fig Three-primer genotyping of malformed embryos and normal siblings indicates linkage of the phenotype to *aldh1a2* exon 8 deletion. **a**. diagram of the locus. **b**. genotyping of 3 dpf embryos displaying heart and pectoral fin defects. **c**. genotyping of embryos after *in situ* hybridization for *tbx18*. All 6 embryos lacking *tbx18* expression in the pectoral fin buds were homozygous for the exon 8 deletion. All siblings that were positive for *tbx18* in the pectoral fin buds were either heterozygous or homozygous wild type.
(PDF)

S9 Fig Germline-transmitted integrations of the loxP site into intron 7 of *aldh1a2*. **a**. All three batches of 20 embryos from R(3x4) family were positive by 5’ and 3’ nested PCR. Sequencing of these PCR fragments revealed an addition of 17 base pairs immediately 3’ of the loxP site, at the 5’ end of the HDR template oligo (See Fig 4). Analysis at the single F1 embryo level was not performed. **b**. All three batches of 20 embryos from R(7x8) family were positive by 5’ and 3’ nested PCR, and 4/20 embryos contained a larger band by flanking PCR. Sequencing revealed an addition of 46 base pairs within the 5’ homology arm, encompassing partial sequence duplication. **c**. All three batches of 20 embryos from R(11x12) pair were positive by 5’ and 3’ nested PCR, and 4/8 embryos contained a larger band by flanking PCR. Sequencing revealed an addition of 19 base pairs 3’ of the loxP site (within the 5’ homology arm). **d**. All three batches of 20 embryos from R(13x14) pair were positive by 5’ and 3’ nested PCR, and 1/8 embryos contained a larger band by flanking PCR. Sequencing revealed presence of almost perfect integration of the loxP site, with a single nucleotide substitution within the 5’ homology arm. **e**. Tail clips of adult F1s from R(9x10) and R(13x14) families leading to establishment of in *aldh1a2*^*tpl139*^ and *aldh1a2*^*tpl140*^ loxP integration lines.
(PDF)

S10 Fig Testing of two sgRNAs targeting 5’ UTR of *tcf21*. **a**. Diagram of the *tcf21* locus with sequence of *tcf21* sgRNA5 and *tcf21* sgRNA6 targets shown in blue. **b**. Sequencing of PCR fragment obtained on a bulk genomic DNA from a pool of 20 embryos injected with *tcf21* sgRNA5 nCas9n mRNA. Direction of the sequencing reaction is shown by black arrow above, PAM motif is bold, sgRNA target blue and the expected Cas9 cut site is indicated by red arrows. **c**. Identical experiment testing the activity of *tcf21* sgRNA6.
(PDF)

S11 Fig Integration of loxP site into 5’ UTR of *tcf21*. a. Diagram of the *tcf21* locus. Both exons and the intron are drawn to scale. Reading frame phase is indicated below each intron-exon junction. b. *tcf21* sgRNA5 target site in the 5’ UTR and HDR oligonucleotide used to knock in the loxP site. c. Sequence of the recovered *tcf21*^*tpl144*^ loxP-containing allele. Single nucleotide substitution within the 5’ homology arm is highlighted in bold red.
(PDF)

S1 Table *tbx20* excision qPCR data. qPCR data analysis of excision efficiency from 5 assays. **Sheet 1**. Not Treated, 5 μM 4-HT at 6hpf, 5 μM 4-HT at 10 hpf. **Sheet 2**. Not treated, μM 4-HT at 6 hpf and 10 hpf, and *tbx20*^*tpl145***Δ/Δ**^. **Sheet 3**. Dose response assay in *ubb*:*CreERT2*.C. **Sheet 4**. Dose response assay in *ubb*:*CreERT2*.*F*. **Sheet 5**. 4-HT treatment at various timepoints, doses, and in both ‘C’ and ‘F’ driver lines as described in Fig 2(l).
(XLSX)

S2 Table Survival rates of embryos treated with varying doses of 4-HT at 6 hpf and 10 hpf. (XLSX)

S3 Table Germline transmission of loxP integrations. In HDR oligo design column, oligonucleotides are diagrammed 5’ to 3’, with homology arms in magenta, spacers in grey and loxP sites in aqua. S indicates oligonucleotides sense to PAM, AS indicates antisense to PAM. * indicates that HDR oligonucleotide template did not match the target site perfectly but had one-nucleotide indel, likely leading to lower germline transmission rates.
(PDF)

S4 Table Sequences of primers used in this study. loxP sites are highlighted in blue and spacers highlighted in grey. sgRNA target site sequences are highlighted in yellow, with the additional G added for T7 transcription shown in bold red. In HDR primers, the loxP site is highlighted in aqua and the spacer sequence is highlighted in grey.
(PDF)
