# Supplementary material for: Conditional mutagenesis by oligonucleotide-mediated integration of loxP sites in zebrafish
Source: PLoS Genet. 2018 Nov 14;14(11):e1007754. doi: 10.1371/journal.pgen.1007754 (PMC6261631; doi:10.1371/journal.pgen.1007754)
Supplement: S1 Fig — a. Diagram of the tbx20 locus around tbx20sgRNA9 cut site. Underneath, expected fragment sizes after digestion of T7 endonuclease or BspLI restriction endonuclease. b. The BspLI restriction enzyme site overlaps the expected double strand break site, indicating that almost all indels will result in loss of BspLI site. c. Gel electrophoresis of PCR fragments digested with T7 Endonuclease or BspLI restriction endonuclease. (PDF) [file pgen.1007754.s001.pdf]

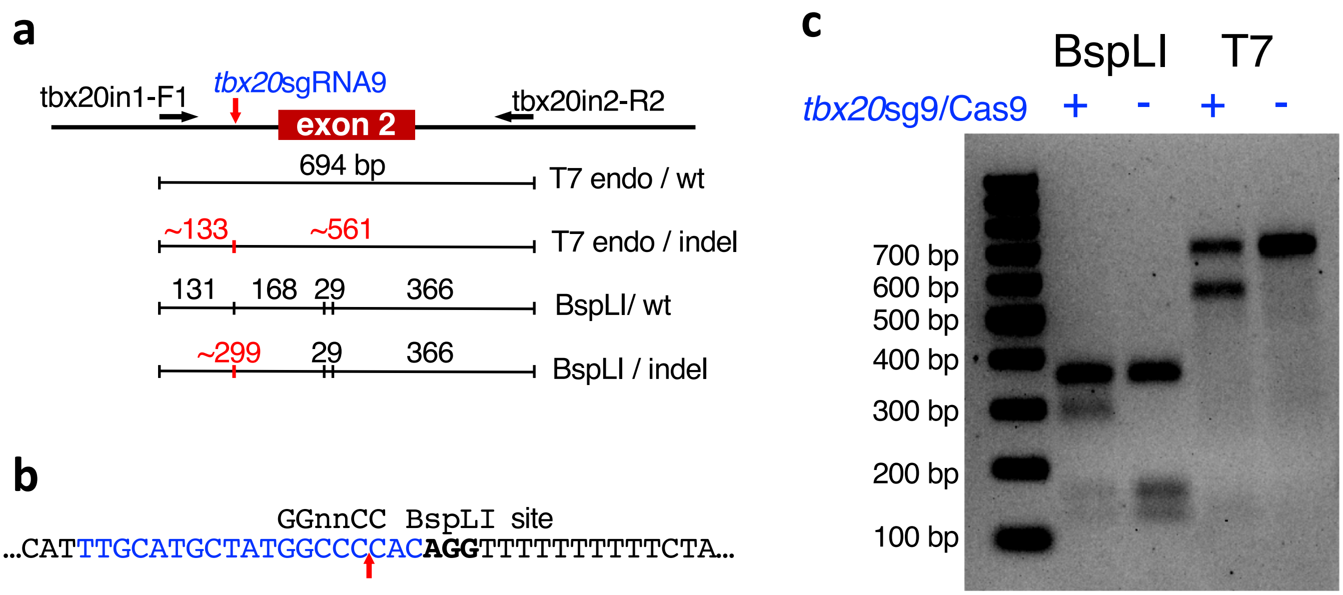

Supplementary Figure 1. Assessment of guide RNA activity by T7 endonuclease assay and by loss of a restriction enzyme site. a. Diagram of the tbx20 locus around tbx20sgRNA9 cut site. Underneath, expected fragment sizes after digestion of T7 endonuclease or BspLI restriction endonuclease. **b.** The BspLI restriction enzyme site overlaps the expected double strand break site, indicating that almost all indels will result in loss of BspLI site. **c.** Gel electrophoresis of PCR fragments digested with T7 Endonuclease or BspLI restriction endonuclease.
