# Supplementary material for: Conditional mutagenesis by oligonucleotide-mediated integration of loxP sites in zebrafish
Source: PLoS Genet. 2018 Nov 14;14(11):e1007754. doi: 10.1371/journal.pgen.1007754 (PMC6261631; doi:10.1371/journal.pgen.1007754)
Supplement: S2 Fig — a, b. Sequencing of individual embryos from outcross of candidate founder #NP29. One out of sixteen embryos contained an incomplete loxP site with a small deletion (a), and two out of sixteen embryos were heterozygous for an allele with a complete loxP site containing a single nucleotide substitution, with an insertion of additional 64 nucleotides (b). c. Sequencing of individual embryos from cross of founder #NP33. Nine out of sixteen individual embryos were heterozygous for this integration of an incomplete loxP site. d. Sequencing of individual embryo from cross of founder #NP39. Five out of sixteen individual embryos were heterozygous for integration of a complete loxP site with an insertion of additional 62 nucleotides. Floxed allele tbx20tpl145 (Fig 2) was established from this founder. e, f. Sequencing of individual embryos from outcross of founder #NP58. Four out of sixteen individual embryos were heterozygous for an integration of an incomplete loxP site with insertion of additional 21 nucleotides (e), and one out of sixteen was heterozygous for integration of complete loxP site containing a single nucleotide substitution, with insertions of 16 and 55 nucleotides on each side of the loxP site (f). Note that all large insertions appear to be partial target site duplications. (PDF) [file pgen.1007754.s002.pdf]

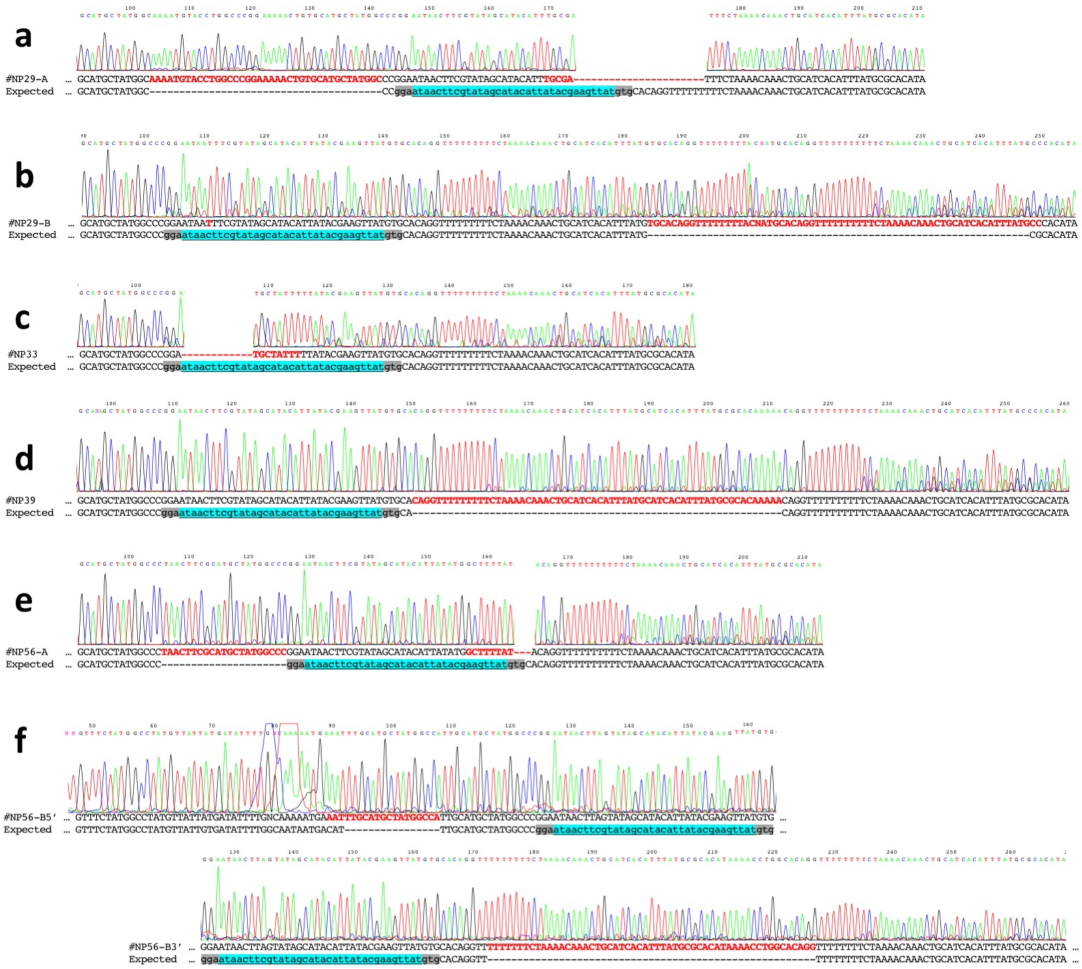

Supplementary Figure 2. Screening for integrations of the second loxP site into *tbx20*. a, b. Sequencing of individual embryos from outcross of candidate founder #NP29. One out of sixteen embryos contained an incomplete loxP site with a small deletion (**a**), and two out of sixteen embryos were heterozygous for an allele with a complete loxP site containing a single nucleotide substitution, with an insertion of additional 64 nucleotides (**b**). **c.** Sequencing of individual embryos from cross of founder #NP33. Nine out of sixteen individual embryos were heterozygous for this integration of an incomplete loxP site. **d.** Sequencing of individual embryo from cross of founder #NP39. Five out of sixteen individual embryos were heterozygous for integration of a complete loxP site with an insertion of additional 62 nucleotides. Floxed allele $tbx20^{tpl145}$ (**Figure 2**) was established from this founder. **e, f.** Sequencing of individual embryos from outcross of founder #NP58. Four out of sixteen individual embryos were heterozygous for an integration of an incomplete loxP site with insertion of additional 21 nucleotides (**e**), and one out of sixteen was heterozygous for integration of complete loxP site containing a single nucleotide substitution, with insertions of 16 and 55 nucleotides on each side of the loxP site (**f**). Note that all large insertions appear to be partial target site duplications.
