# Supplementary material for: Conditional mutagenesis by oligonucleotide-mediated integration of loxP sites in zebrafish
Source: PLoS Genet. 2018 Nov 14;14(11):e1007754. doi: 10.1371/journal.pgen.1007754 (PMC6261631; doi:10.1371/journal.pgen.1007754)
Supplement: S3 Fig — a. Experimental design. Fish homozygous for the floxed allele are incrossed, and half the embryos are injected with Cre mRNA. b-e. Representative images of Cre-injected (b,d) and un-injected siblings (c,e) at 1 dpf (b,c) and 3 dpf (d,e). (PDF) [file pgen.1007754.s003.pdf]

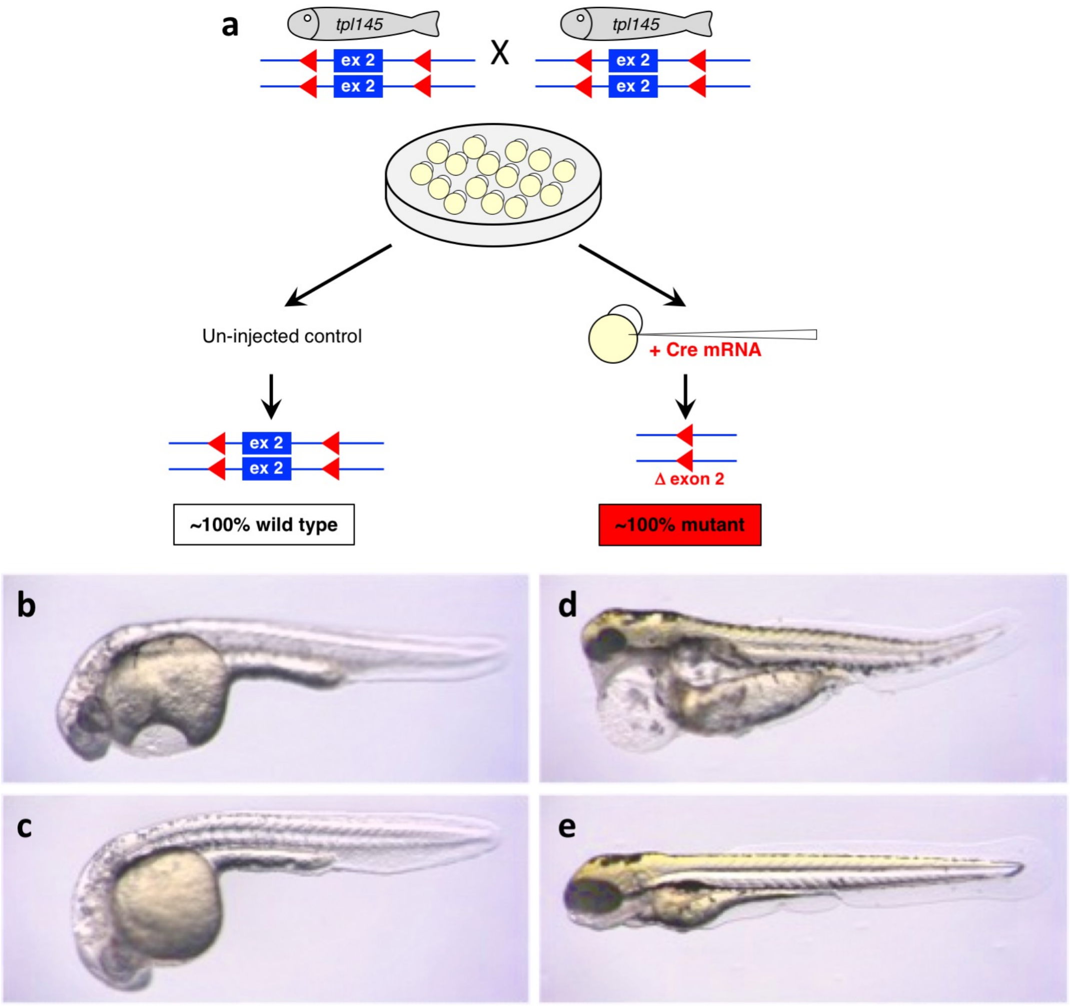

Supplementary Figure 3. Generation of "all-mutant" clutches of embryos. a. Experimental design. Fish homozygous for the floxed allele are incrossed, and half the embryos are injected with Cre mRNA. **b-e.** Representative images of Cre-injected (**b,d**) and un-injected siblings (**c,e**) at 1 dpf (**b,c**) and 3 dpf (**d,e**).
