# Supplementary material for: Conditional mutagenesis by oligonucleotide-mediated integration of loxP sites in zebrafish
Source: PLoS Genet. 2018 Nov 14;14(11):e1007754. doi: 10.1371/journal.pgen.1007754 (PMC6261631; doi:10.1371/journal.pgen.1007754)
Supplement: S4 Fig — tpl122 contains a 241 bp deletion removing most of exon 2. tpl122 homozygotes display a consistent and strong tbx20 phenotype. (PDF) [file pgen.1007754.s004.pdf]

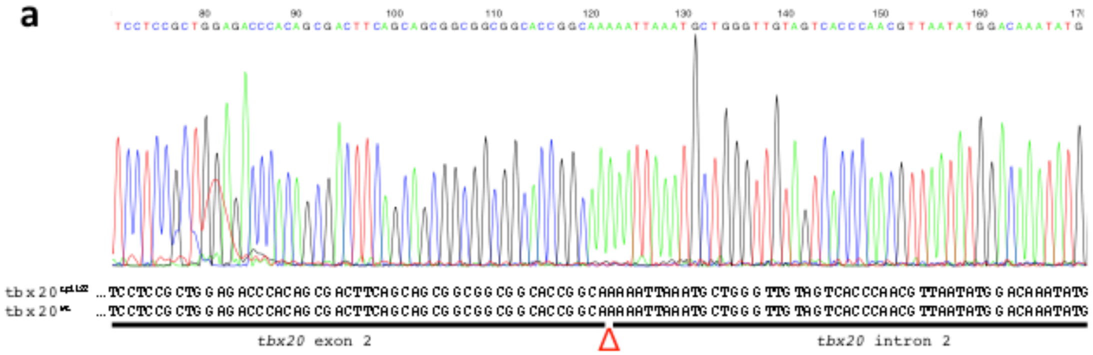

Supplementary Figure 4. Sequence of *tbx20*^{tpl122} partial deletion allele. *tpl122* contains a 241 bp deletion removing most of exon 2 and part of intron 2. *tpl122* homozygotes display a consistent and strong *tbx20* phenotype.
