# Supplementary material for: Conditional mutagenesis by oligonucleotide-mediated integration of loxP sites in zebrafish
Source: PLoS Genet. 2018 Nov 14;14(11):e1007754. doi: 10.1371/journal.pgen.1007754 (PMC6261631; doi:10.1371/journal.pgen.1007754)
Supplement: S5 Fig — a. Embryos were treated with 5 μM 4-HT at either 6 hpf or 10 hpf. Embryos were pooled (n = 20) and collected at 30, 60, 120, and 240 minutes after exposure. 0 indicates a pool of siblings not exposed to 4-HT. Note: 6 hpf and 10 hpf not treated control PCRs were performed on the same DNA sample. (PDF) [file pgen.1007754.s005.pdf]

a

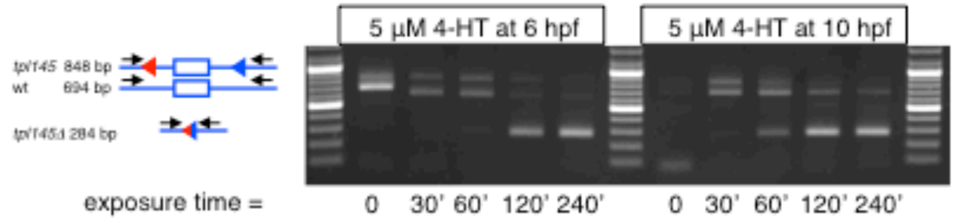

Supplementary Figure 5. Time course of excision after 4-HT exposure. a. Embryos were treated with 5 µM 4-HT at either 6 hpf or 10 hpf. Embryos were pooled (n=20) and collected at 30, 60, 120, and 240 minutes after exposure. 0 indicates a pool of siblings not exposed to 4-HT. Note: 6 hpf and 10 hpf not treated control PCRs were performed on the same DNA sample.
