# Supplementary material for: Conditional mutagenesis by oligonucleotide-mediated integration of loxP sites in zebrafish
Source: PLoS Genet. 2018 Nov 14;14(11):e1007754. doi: 10.1371/journal.pgen.1007754 (PMC6261631; doi:10.1371/journal.pgen.1007754)
Supplement: S6 Fig — Expression of retinoic acid synthesizing genes (aldh1a2 and aldh8a1) in adult zebrafish hearts at various time points post sham operation or cardiac injury in fragments per kilobase of transcript per million mapped reads (FPKM). aldh1a2 was highly upregulated in response to injury. aldh1a3 was not detectable at any tested time point. hps, hours post sham injury, hpi, hours post injury, dps, days post sham injury, dpi, days post injury. (PDF) [file pgen.1007754.s006.pdf]

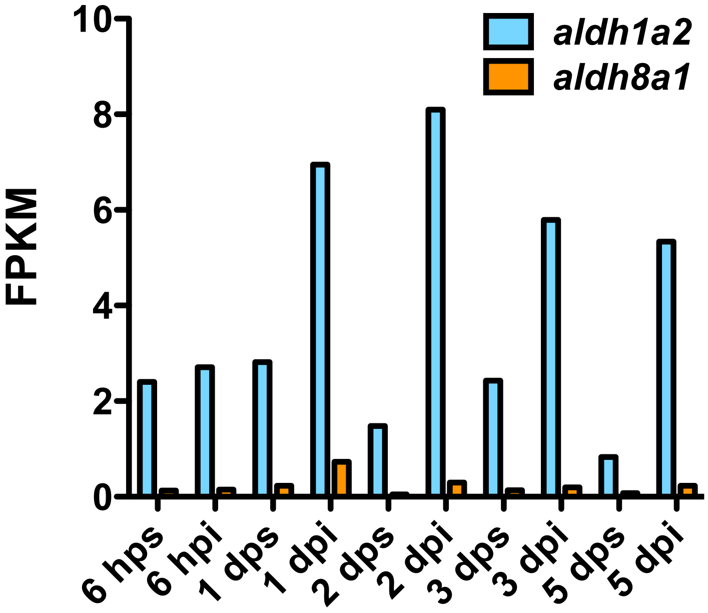

Supplementary Figure 6. Expression on RA-synthesizing enzymes in response to heart injury. Expression of retinoic acid synthesizing genes (*aldh1a2 and aldh8a1*) in adult zebrafish hearts at various time points post sham operation or cardiac injury in fragments per kilobase of transcript per million mapped reads (FPKM). *aldh1a2* was highly upregulated in response to injury. *aldh1a3* was not detectable at any tested time point. hps, hours post sham injury, hpi, hours post injury, dps, days post sham injury, dpi, days post injury.
