# Supplementary material for: Conditional mutagenesis by oligonucleotide-mediated integration of loxP sites in zebrafish
Source: PLoS Genet. 2018 Nov 14;14(11):e1007754. doi: 10.1371/journal.pgen.1007754 (PMC6261631; doi:10.1371/journal.pgen.1007754)
Supplement: S7 Fig — a. Sequencing of PCR fragments amplified from bulk DNA obtained from pools of 6–10 embryos injected with aldh1a2 sgRNA1, aldh1a2 sgRNA2, aldh1a2 sgRNA3 and aldh1a2 sgRNA4 along with nCas9n mRNA. The sequences are in 5’ -> 3’ with regard to aldh1a2 locus. Sequences corresponding to single guide RNA are shown in blue, PAM motifs are bold, and expected Cas9 cut sites are indicated by red arrows. Direction of the sequencing reaction is shown by black arrows above. b. Detection of exon 8 deletions in DNA from pools of 20 embryos from F0 incrosses A (ΔinxA) and B (ΔinxB). Diagram of the locus on top corresponds to gel electrophoresis results on the botttom, with a yellow arrow pointing to the expected deletion band. c. PCR genotyping of adult F1 fish. Two out of 17 F1 fish from ΔinxA family carry a deletion, and one out of 15 F1 fish from the ΔinxB family carries a deletion. d. Sequencing of aldh1a2tpl137 deletion allele recovered from ΔinxA family. e. Sequencing of aldh1a2tpl138 deletion allele recovered from ΔinxB family. (PDF) [file pgen.1007754.s007.pdf]

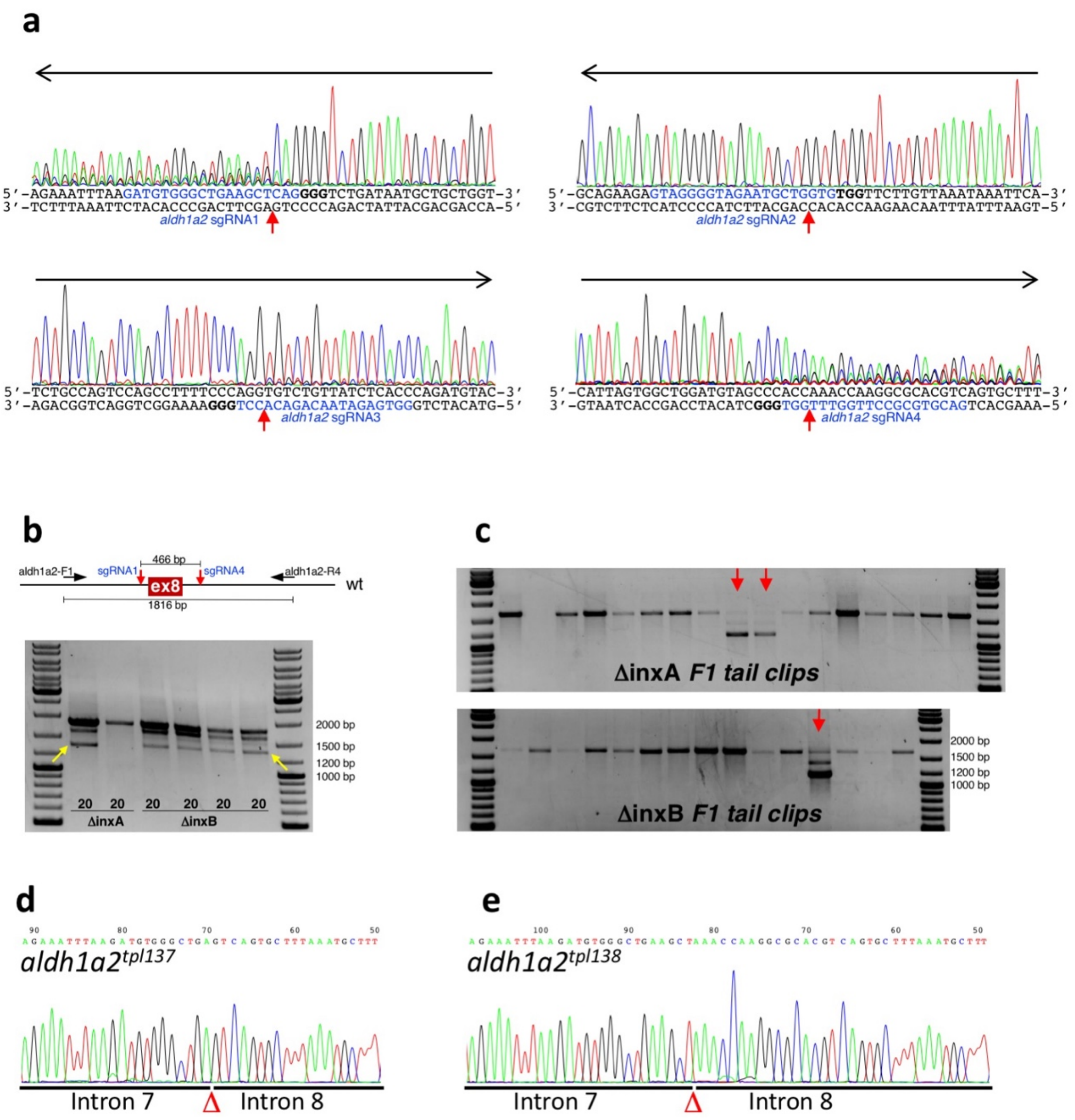

Supplementary Figure 7. Testing of sgRNAs targeting *aldh1a2* and isolation of exon 8 deletion lines. a. Sequencing of PCR fragments amplified from bulk DNA obtained from pools of 6-10 embryos injected with *aldh1a2* sgRNA1, *aldh1a2* sgRNA2, *aldh1a2* sgRNA3 and *aldh1a2* sgRNA4 along with nCas9n mRNA. The sequences are in 5' -> 3' with regard to *aldh1a2* locus. Sequences corresponding to single guide RNA are shown in blue, PAM motifs are bold, and expected Cas9 cut sites are indicated by red arrows. Direction of the sequencing reaction is shown by black arrows above. **b.** Detection of exon 8 deletions in DNA from pools of 20 embryos from F0 incrosses A (ΔinxA) and B (ΔinxB). Diagram of the locus on top corresponds to gel electrophoresis results on the botttom, with a yellow arrow pointing to the expected deletion band. **c.** PCR genotyping of adult F1 fish. Two out of 17 F1 fish from ΔinxA family carry a deletion, and one out of 15 F1 fish from the ΔinxB family carries a deletion. **d.** Sequencing of *aldh1a2*^{tpl137} deletion allele recovered from ΔinxA family. **e.** Sequencing of *aldh1a2*^{tpl138} deletion allele recovered from ΔinxB family.
