# Supplementary material for: Conditional mutagenesis by oligonucleotide-mediated integration of loxP sites in zebrafish
Source: PLoS Genet. 2018 Nov 14;14(11):e1007754. doi: 10.1371/journal.pgen.1007754 (PMC6261631; doi:10.1371/journal.pgen.1007754)
Supplement: S8 Fig — a. diagram of the locus. b. genotyping of 3 dpf embryos displaying heart and pectoral fin defects. c. genotyping of embryos after in situ hybridization for tbx18. All 6 embryos lacking tbx18 expression in the pectoral fin buds were homozygous for the exon 8 deletion. All siblings that were positive for tbx18 in the pectoral fin buds were either heterozygous or homozygous wild type. (PDF) [file pgen.1007754.s008.pdf]

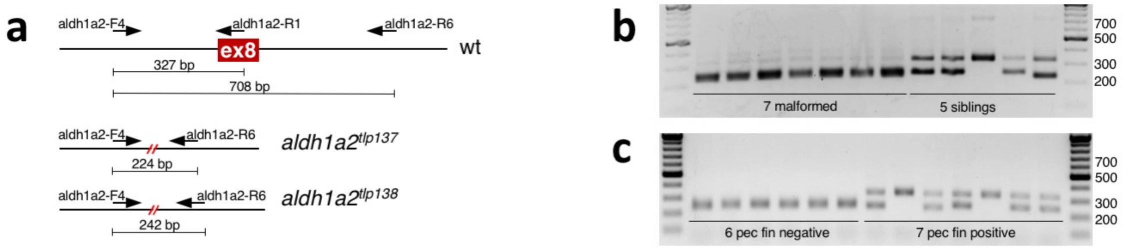

Supplementary Figure 8. Three-primer genotyping of malformed embryos and normal siblings indicates linkage of the phenotype to *aldh1a2* exon 8 deletion. **a**. diagram of the locus. **b**. genotyping of 3 dpf embryos displaying heart and pectoral fin defects. **c**. genotyping of embryos after *in situ* hybridization for *tbx18*. All 6 embryos lacking *tbx18* expression in the pectoral fin buds were homozygous for the exon 8 deletion. All siblings that were positive for *tbx18* in the pectoral fin buds were either heterozygous or homozygous wild type.
