# Supplementary material for: Conditional mutagenesis by oligonucleotide-mediated integration of loxP sites in zebrafish
Source: PLoS Genet. 2018 Nov 14;14(11):e1007754. doi: 10.1371/journal.pgen.1007754 (PMC6261631; doi:10.1371/journal.pgen.1007754)
Supplement: S9 Fig — a. All three batches of 20 embryos from R(3x4) family were positive by 5’ and 3’ nested PCR. Sequencing of these PCR fragments revealed an addition of 17 base pairs immediately 3’ of the loxP site, at the 5’ end of the HDR template oligo (See Fig 4). Analysis at the single F1 embryo level was not performed. b. All three batches of 20 embryos from R(7x8) family were positive by 5’ and 3’ nested PCR, and 4/20 embryos contained a larger band by flanking PCR. Sequencing revealed an addition of 46 base pairs within the 5’ homology arm, encompassing partial sequence duplication. c. All three batches of 20 embryos from R(11x12) pair were positive by 5’ and 3’ nested PCR, and 4/8 embryos contained a larger band by flanking PCR. Sequencing revealed an addition of 19 base pairs 3’ of the loxP site (within the 5’ homology arm). d. All three batches of 20 embryos from R(13x14) pair were positive by 5’ and 3’ nested PCR, and 1/8 embryos contained a larger band by flanking PCR. Sequencing revealed presence of almost perfect integration of the loxP site, with a single nucleotide substitution within the 5’ homology arm. e. Tail clips of adult F1s from R(9x10) and R(13x14) families leading to establishment of in aldh1a2tpl139 and aldh1a2tpl140 loxP integration lines. (PDF) [file pgen.1007754.s009.pdf]

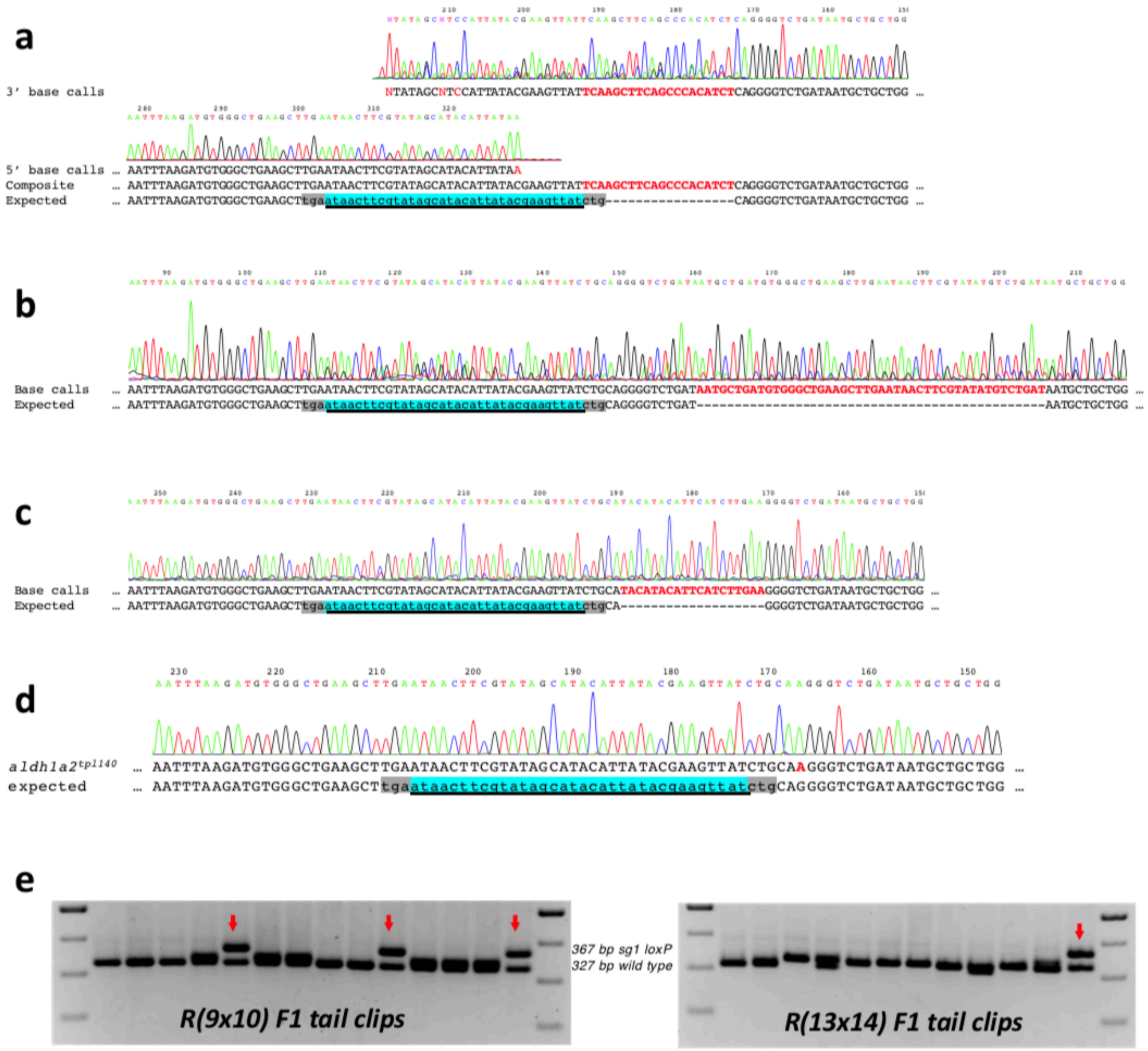


**Supplementary Figure 9. Germline-transmitted integrations of the loxP site into intron 7 of *aldh1a2*. a.** All three batches of 20 embryos from R(3x4) family were positive by 5' and 3' nested PCR. Sequencing of these PCR fragments revealed an addition of 17 base pairs immediately 3' of the loxP site, at the 5' end of the HDR template oligo (See **Figure 4**). Analysis at the single F1 embryo level was not performed. **b.** All three batches of 20 embryos from R(7x8) family were positive by 5' and 3' nested PCR, and 4/20 embryos contained a larger band by flanking PCR. Sequencing revealed an addition of 46 base pairs within the 5' homology arm, encompassing partial sequence duplication. **c.** All three batches of 20 embryos from R(11x12) pair were positive by 5' and 3' nested PCR, and 4/8 embryos contained a larger band by flanking PCR. Sequencing revealed an addition of 19 base pairs 3' of the loxP site (within the 5' homology arm). **d.** All three batches of 20 embryos from R(13x14) pair were positive by 5' and 3' nested PCR, and 1/8 embryos contained a larger band by flanking PCR. Sequencing revealed presence of almost perfect integration of the loxP site, with a single nucleotide substitution within the 5' homology arm. **e.** Tail clips of adult F1s from R(9x10) and R(13x14) families leading to establishment of in *aldh1a2*$^{tpl139}$ and *aldh1a2*$^{tpl140}$ loxP integration lines.
