# Supplementary material for: Conditional mutagenesis by oligonucleotide-mediated integration of loxP sites in zebrafish
Source: PLoS Genet. 2018 Nov 14;14(11):e1007754. doi: 10.1371/journal.pgen.1007754 (PMC6261631; doi:10.1371/journal.pgen.1007754)
Supplement: S10 Fig — a. Diagram of the tcf21 locus with sequence of tcf21 sgRNA5 and tcf21 sgRNA6 targets shown in blue. b. Sequencing of PCR fragment obtained on a bulk genomic DNA from a pool of 20 embryos injected with tcf21 sgRNA5 nCas9n mRNA. Direction of the sequencing reaction is shown by black arrow above, PAM motif is bold, sgRNA target blue and the expected Cas9 cut site is indicated by red arrows. c. Identical experiment testing the activity of tcf21 sgRNA6. (PDF) [file pgen.1007754.s010.pdf]

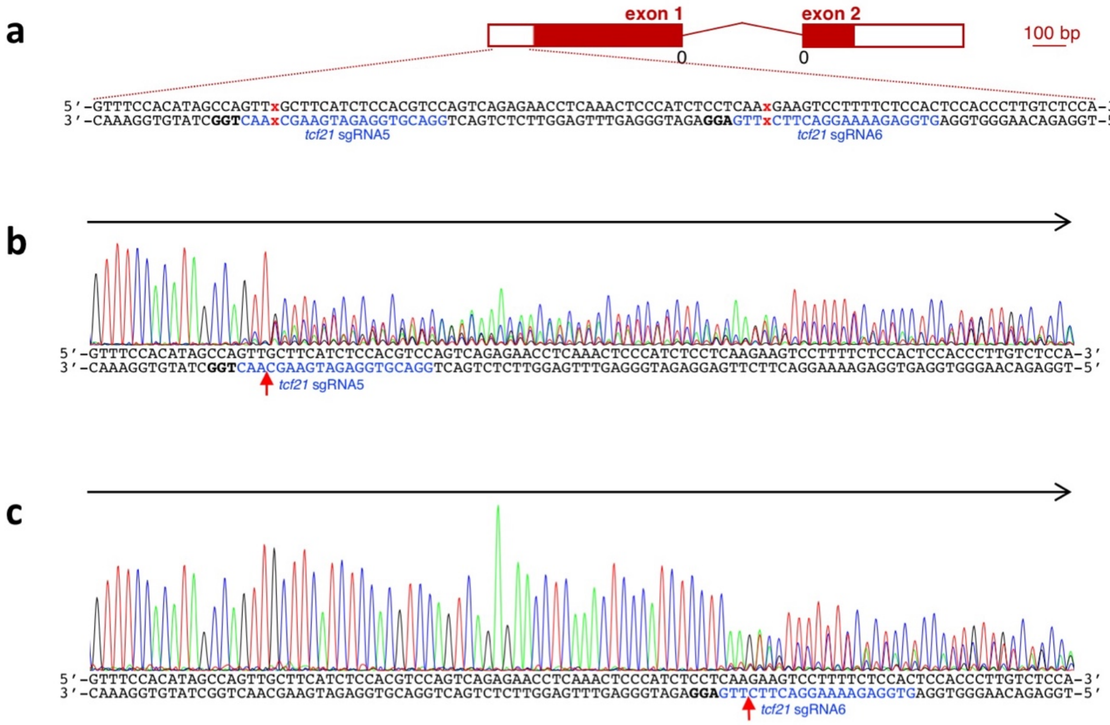

Supplementary Figure 10. Testing of two sgRNAs targeting 5' UTR of *tcf21*. a. Diagram of the *tcf21* locus with sequence of *tcf21* sgRNA5 and *tcf21* sgRNA6 targets shown in blue. **b**. Sequencing of PCR fragment obtained on a bulk genomic DNA from a pool of 20 embryos injected with *tcf21* sgRNA5 nCas9n mRNA. Direction of the sequencing reaction is shown by black arrow above, PAM motif is bold, sgRNA target blue and the expected Cas9 cut site is indicated by red arrows. **c.** Identical experiment testing the activity of *tcf21* sgRNA6.
