# Supplementary material for: Conditional mutagenesis by oligonucleotide-mediated integration of loxP sites in zebrafish
Source: PLoS Genet. 2018 Nov 14;14(11):e1007754. doi: 10.1371/journal.pgen.1007754 (PMC6261631; doi:10.1371/journal.pgen.1007754)
Supplement: S11 Fig — a. Diagram of the tcf21 locus. Both exons and the intron are drawn to scale. Reading frame phase is indicated below each intron-exon junction. b. tcf21 sgRNA5 target site in the 5’ UTR and HDR oligonucleotide used to knock in the loxP site. c. Sequence of the recovered tcf21tpl144 loxP-containing allele. Single nucleotide substitution within the 5’ homology arm is highlighted in bold red. (PDF) [file pgen.1007754.s011.pdf]

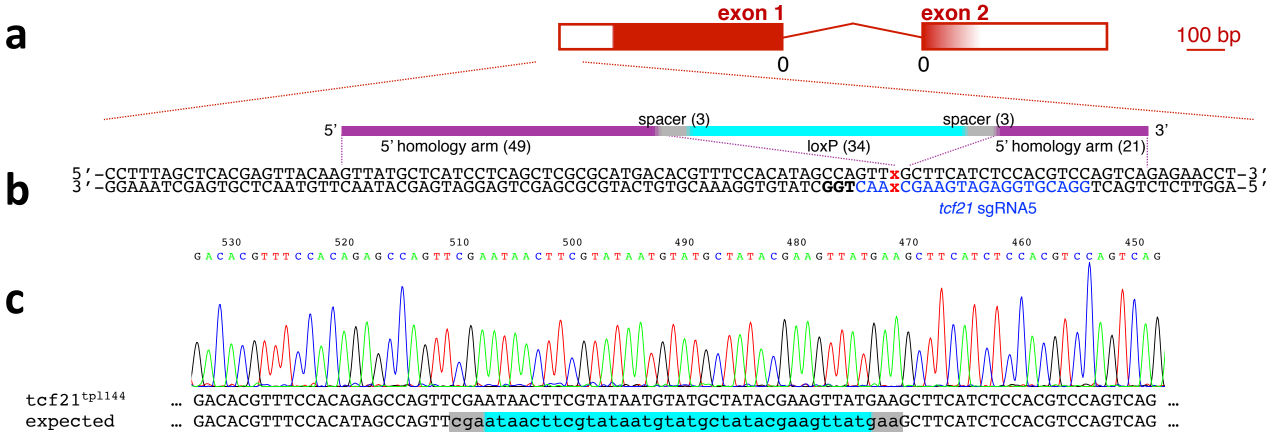

Supplementary Figure 11. Integration of loxP site into 5’ UTR of *tcf21*. a. Diagram of the *tcf21* locus. Both exons and the intron are drawn to scale. Reading frame phase is indicated below each intron-exon junction. **b.** *tcf21* sgRNA5 target site in the 5’ UTR and HDR oligonucleotide used to knock in the loxP site. **c.** Sequence of the recovered *tcf21*^{tpl144} loxP-containing allele. Single nucleotide substitution within the 5’ homology arm is highlighted in bold red.
