# Supplementary material for: Conditional mutagenesis by oligonucleotide-mediated integration of loxP sites in zebrafish
Source: PLoS Genet. 2018 Nov 14;14(11):e1007754. doi: 10.1371/journal.pgen.1007754 (PMC6261631; doi:10.1371/journal.pgen.1007754)
Supplement: S3 Table — In HDR oligo design column, oligonucleotides are diagrammed 5’ to 3’, with homology arms in magenta, spacers in grey and loxP sites in aqua. S indicates oligonucleotides sense to PAM, AS indicates antisense to PAM. * indicates that HDR oligonucleotide template did not match the target site perfectly but had one-nucleotide indel, likely leading to lower germline transmission rates. (PDF) [file pgen.1007754.s014.pdf]

**Target**	HDR oligo design	Germline transmission	Germline mosaicism	Allele description	Line designation
***tbx20* intron 2**	21-3-loxP-3-21 S	**1 / 24**	**4 / 16** adult F1	+10	*tpl135*
***tbx20* intron 1**	62-3-loxP-3-18 AS	**1 / 6**	**3 / 16** adult F1	Precise	*tpl136*
***tbx20* intron 1**	62-3-loxP-3-18 AS	**1 / 42***	**4 / 8** adult F1	+62	*tpl145*
***fleer* intron 7**	49-3-loxP-3-21 AS	**1 / 14**	**1 / 21** adult F1	Precise	*tpl141*
***aldh1a2* intron 7**	49-3-loxP-3-21 AS	**5 / 14**	**3 / 14** adult F1<br>**6 / 16** adult F1<br>nd<br>**4 / 24** @5 dpf<br>**5 / 8** @5 dpf	Precise<br>1 SNP<br>-3 +20<br>+46<br>+19	*tpl139*<br>*tpl140*<br>-<br>-<br>-
***tcf21* 5' UTR**	49-3-loxP-3-21 AS	**1 / 18**	**4 / 24** adult	Precise	*tpl144*

Supplementary Table 3. Germline transmission of loxP integrations. In HDR oligo design column, oligonucleotides are diagrammed 5' to 3', with homology arms in magenta, spacers in grey and loxP sites in aqua. S indicates oligonucleotides sense to PAM, AS indicates antisense to PAM. * indicates that HDR oligonucleotide template did not match the target site perfectly but had one-nucleotide indel, likely leading to lower germline transmission rates.
