# Supplementary material for: Conditional mutagenesis by oligonucleotide-mediated integration of loxP sites in zebrafish
Source: PLoS Genet. 2018 Nov 14;14(11):e1007754. doi: 10.1371/journal.pgen.1007754 (PMC6261631; doi:10.1371/journal.pgen.1007754)
Supplement: S4 Table — loxP sites are highlighted in blue and spacers highlighted in grey. sgRNA target site sequences are highlighted in yellow, with the additional G added for T7 transcription shown in bold red. In HDR primers, the loxP site is highlighted in aqua and the spacer sequence is highlighted in grey. (PDF) [file pgen.1007754.s015.pdf]

Supplementary Table 4. Sequences of primers used in this study. loxP sites are highlighted in blue and spacers highlighted in grey. sgRNA target site sequences are highlighted in yellow, with the additional G added for T7 transcription shown in bold red. In HDR primers, the loxP site is highlighted in aqua and the spacer sequence is highlighted in grey.

Primer	Sequence 5′ to 3′
**universal primers for sgRNA synthesis** (from Burg *et al*., 2016)	
sgT7	GCTAGCTAATACGACTCACT
sgRNA-R	AAAAGCACCGACTCGGTG
M13F	GTAAAACGACGGCCAGT
gene specific primers for sgRNA synthesis	
*tbx20*sgRNA9	CGCTAGCTAATACGACTCACTATA**g**ttgcatgctatggccccac<br>GTTTTAGAGCTAGAAATAG
*tbx20*sgRNA10	CGCTAGCTAATACGACTCACTATAgttgtcacccaacgttaata<br>GTTTTAGAGCTAGAAATAG
*flr*sgRNA3 (gBlock, IDT)	ccGCTAGCTAATACGACTCACTATAgggttcataagaagagtctg<br>GTTTTAGAGCTAGAAATAGCAAGTTAAAATAAGGCTAGTCCGTTA<br>TCAACTTGAAAAAGTGGCACCGAGTCGGTGCTTTT
*aldh1a2*sgRNA1	CGCTAGCTAATACGACTCACTATAgatgtgggctgaagctcag<br>GTTTTAGAGCTAGAAATAG
*aldh1a2*sgRNA2	CGCTAGCTAATACGACTCACTATAgtaggggtagaatgctggtg<br>GTTTTAGAGCTAGAAATAG
*aldh1a2*sgRNA3	CGCTAGCTAATACGACTCACTATAggtgagataacagacacct<br>GTTTTAGAGCTAGAAATAG
*aldh1a2*sgRNA4	CGCTAGCTAATACGACTCACTATAgacgtgcgccttggtttggt<br>GTTTTAGAGCTAGAAATAG
*tcf21*sgRNA5	CGCTAGCTAATACGACTCACTATAggacgtggagatgaagcaac<br>GTTTTAGAGCTAGAAATAG
*tcf21*sgRNA6	CGCTAGCTAATACGACTCACTATAggtggagaaaggacttcttg<br>GTTTTAGAGCTAGAAATAG
**gene specific loxP HDR oligos**	
tbx20sg10-loxP-F1	GGTTGTTGTCACCCAACGTTAggaataacttcgtatagcatacat<br>tatacgaagttattgcATATGGACAAATATGGATAAA
tbx20sg9-loxP-103	CAATAACCATGATTATGTGCGCATAAATGTGATGCAGTTTGTTTT<br>AGAAAAAAAAACCTGTGcacataacttcgtataatgtatgctata<br>cgaagttattccGGGCCATAGCATGCAAAT
flrup3-loxP-HDR3	AGAAGTTGCATTTAAATCAAATGCATAAACGAATCCTCGAAGGCC<br>ACAGctgataacttcgtatagcatacattatacgaagttattgtA<br>CTCTTCTTATGAACCTGTTT
aldh1a2sg1loxP-hdrL	TCGTCCCCTGCTCTTTGACTGGACAACCAGCAGCATTATCAGaCC<br>CCTGcagataacttcgtataatgtatgctatacgaagttattcaA<br>GCTTCAGCCCACATCTTAAA
tcf21sg5loxP-HDRL	GTTATGCTCATCCTCAGCTCGCGCATGACACGTTTCCACATAGCC<br>AGTTcgaataacttcgtataatgtatgctatacgaagttatgaaG

	CTTCATCTCCACGTCCAGTC
**Tol2 (gene trap) – specific** (from Balciuniene *et al*., 2013)	
Tol2-F8	CTCAAGTAAGATTCATAGCCAGATAC
Tol2-R7	CTGGCTAGAATCTTACTTGAGTAA
**loxP – specific**	
loxP-U1	TATAATGTATGCTATACGAAGTTAT
loxP-U2	TATAGCATACATTATACGAAGTTAT
loxP-F1	TCGTATAATGTATGCTATACGAAGT
loxP-R1	ATAACTTCGTATAGCATACATTATA
loxP-Rev	CGTATAATGTATGCTATACG
***tbx20* – specific**	
tbx20in1-F1	CAGTAGCCTAATATTTCAACGTTTC
tbx20in1-F2	GTTTCTATGGCCTATGTTATTGTGA
tbx20in1-F4	TGTAAAGACTGATGTAGTATGTAGT
tbx20in2-R1	GTGTTGACAGAAATAGGTTACACTC
tbx20in2-R2	GTAAGCTTGTGAGATTGTGTTGGAC
tbx20ex2-F1	AAATGATTATTACAAAGTCTGGAAG
tbx20ex2-R1	ACGACTTTTCCACGAATTGTTCTTG
***fleer* – specific**	
flrUTR-F1	GCGTCATGTATTGATTACGTCACT
flrEx7-F1	TCATCCTGCACGAGACTGCT
flrEx7-F2	TAATCTGAAAGCAGCTATTGAATAC
flrIn7-R1	TTGCAATGTTTCCACTGACCAA
flrIn7-R2	AACATGAGACAGAGTGATGTTAAG
flrIn7-R4	TAGACTGCACACAGGCAGACTTGAT
***aldh1a2* – specific**	
aldh1a2-F1	TGCACTGTTAAACAGCGCGTAAGAC
aldh1a2-F2	AAGCTGATCCAAGAAGCAGCAGGAA
aldh1a2-F3	TAACATTAGGCAGGCAGAAGAGTAG
aldh1a2-F4	TGACAGCGTCGAGCATCCTACACAA
aldh1a2-F5	GTTGTAATCGCACATCGGCA
aldh1a2-R1	CTCCAGCGTGACTCTCTTCAGATT
aldh1a2-R4	AGTTTTGGAGCCATTGAATTCTGCT
aldh1a2-R6	AGCAGGCAGATTTACGGTTCTAGTG
aldh1a2-R7	CGGTGAGGGCATCAGATGGA
***tcf21* – specific**	
tcf21-F1	ACAAGTTATGCTCATCCTCAGCTCG
tcf21-F9	CTGCTAGCAACAGCATATCCATC
tcf21-F10	GACAAAACGCTCGAGTGCAATGAAT
tcf21UTR-F1	CTCCACGTCCAGTCAGAGAACCTC

tcf21-R1	CATATTTGTCATTGGCGAGTATCTG
tcf21-R5	GCTTTGCTGAGCACGCGCATCCT
tcf21UTR-R2	AACAAAGGCGCGTGACATGA
***tbx18* – specific**	
tbx18UTR-F1	CGTTGACAGACCGATGAGACTGTT
tbx18-R10	TGGAGTGCAGAATGATATGGCC
